# From intention–behavior gap to dietary affordances: an active inference account of sustainable eating

**DOI:** 10.3389/fpsyg.2026.1779626

**Published:** 2026-08-11

**Authors:** Abigail McIntosh, Amy R. Griffiths, Anthony Brennan, Hayley A. Young

**Affiliations:** School of Psychology, Swansea University, Swansea, United Kingdom

**Keywords:** active inference, behavior change, dietary affordance, intention–behavior gap, public health policy, sustainable diets, systems thinking

## Abstract

Faced with the urgent challenges of climate change and environmental degradation, governments, and frameworks like the EAT–Lancet Commission advocate for sustainable diets. Yet a persistent gap remains between individuals’ intentions to eat sustainably and their actual behavior. Current approaches often attribute this gap to deficits in individual attitudes or motivation. In this paper, we challenge that view. We introduce the construct of dietary affordances to describe how opportunities for action emerge from the interplay between individuals and their environments. Drawing on ecological psychology and contemporary active inference accounts of perception and action, we formalize dietary affordance as a relative selection weight shaped by three components: pragmatic value *V*_*i,c*_ (goal alignment), predictive precision *P*_*i,c*_ (enactment reliability), and the policy prior *H*_*i,c*_ (accumulated habit strength). Crucially, value and reliability contribute additively to a reduced-form policy score *S*_*i,c*_, with non-linear threshold-like effects arising from the exponential structure of policy selection and normalization across competing options. This implies that without sufficient reliability, high motivation alone may be insufficient to sustain behavior - and that entrenched habits can dominate even when better alternatives are nominally available. Within this framework, unsustainable eating is reframed not as an individual failure, but as a predictable response to a misaligned affordance field: for many, the safest, most predictable, and most habitual course of action is to choose foods that undermine climate goals. We argue that closing the intention–behavior gap requires shifting the focus of interventions away from individual consumers and toward the institutions that design and govern food environments. We identify specific leverage points within the food system, including pricing, urban design and social protection, that researchers and policymakers can use to reshape the feasible behavioral policy set, ensuring that sustainable habits are not just intended but practically achievable. By reducing the expected risk and uncertainty of sustainable diets and supporting the formation of sustainable habits, policymakers can align these options with the brain’s drive to minimize uncertainty, ensuring that sustainable behavior emerges not through self-regulation, but as the natural outcome of a well-designed system.

## Introduction

In the face of global challenges such as climate change and resource depletion, sustainable food choices and dietary habits have gained prominence in research and government policy (De Schutter et al., 2020; Crippa et al., 2021). Currently, food production contributes to around one-third of global greenhouse gas emissions (Crippa et al., 2021). Policy makers have recognized that unsustainable dietary practices, characterized by the overconsumption of animal-based products and extensive food waste, are significant contributors to environmental degradation (Springmann et al., 2018; Crippa et al., 2021). In response, dietary guidelines increasingly recommend sustainable, plant-based eating patterns to promote human and environmental health (Willett et al., 2019; Rockström et al., 2025). For example, the EAT–Lancet Commission proposed a sustainable diet pattern (the EAT–Lancet diet) and defined pescatarian, vegetarian and vegan variants to support transitions toward this pattern, emphasizing whole grains, fruits, vegetables, legumes, nuts and unsaturated oils, while reducing animal foods (e.g., poultry and seafood) and limiting red and processed meat, sugar, refined grains and starchy vegetables (Willett et al., 2019; Rockström et al., 2025).

While many consumers report an interest in healthy and environmentally sustainable habits (O’Keefe et al., 2016), surveys consistently show a mismatch between their stated beliefs and their actual behavior (ElHaffar et al., 2020). This mismatch is variously described as the intention–behavior gap (Sheeran, 2002), the motivation–behavior gap (Groening et al., 2018) or, in the context of environmental sustainability, the green intention–behavior gap (Frank and Brock, 2018). In recent years, sustainable diet research has expanded, but relatively few studies and reviews have focused explicitly on this intention–behavior gap (Joshi and Rahman, 2015; ElHaffar et al., 2020). This has practical consequences: a recent umbrella review found that global initiatives rely heavily on labeling and information campaigns (Vargas et al., 2025). While these strategies may successfully target consumer *intentions*, they frequently fail to bridge the gap to *behavior* because they do not address the structural constraints that block action (Vargas et al., 2025). Nonetheless, if governments are to meet ambitious net-zero targets, bridging this gap is crucial.

To advance this goal, the current paper adopts a systems approach that addresses a major limitation of traditional theories: their tendency to treat personal and environmental factors as independent, static influences rather than as evolving, mutually dependent processes. We draw on two complementary traditions. Ecological psychology (Gibson, 1977) provides the concept of affordances - possibilities for action that depend jointly on the organism and its surroundings, rather than on either alone (Lobo et al., 2018). This reframes dietary choice as something that emerges from the relationship between a person and their food environment, not from attitudes or motivation in isolation. Active inference (Friston et al., 2012, 2013) provides the selection mechanism: a formal account of how agents choose among available actions by minimizing expected uncertainty and risk relative to their preferences (Friston et al., 2016) (Box 1 Glossary of Terms). Together, these frameworks specify not only that dietary choices depend on what is available and achievable in context, but why particular options come to dominate behavior - and why changing the environment, rather than strengthening individual motivation, may be the more effective route to sustainable eating.

BOX 1 Glossary of terms.

**Table:** 

Glossary	
Sustainable diet	A dietary pattern that supports health while reducing environmental impacts (e.g., greenhouse gas emissions, land and water use) and maintaining nutritional adequacy.
Intention	An intention is a mental representation of a planned future action or outcome (e.g., “eat more plant-based meals”), which can arise from deliberate reflection or habitual processes. Intentions organize and guide behavior but do not guarantee that the intended action will be carried out.
Intention–behavior gap	The discrepancy between what people endorse as their goals or intentions and what they enact in everyday behavior.
Dietary affordance	The set of food-related action possibilities available to a particular individual in a particular context, arising from person–environment interaction, such as time, income, skills, access, infrastructure, and pricing. In active inference terms, dietary affordances define which behavioral policies are feasible for the agent in context. We define a reduced-form policy score *S*_*i,c*_ as: *S*_*i*,*c*_=*V*_*i*,*c*_ + *P*_*i*,*c*_ and formalize the relative affordance weight of option *i* in context *c* as: ${\tilde{A}}_{i,c}=H_{i,c}\cdot \text{exp}\,\left(\gamma_{c}\cdot S_{i,c}\right)$ with selection probability obtained by normalizing across the feasible affordance field *F_c_*: $P\,\left(\pi_{i}|c\right)=\frac{{\tilde{A}}_{i,c}}{\sum_{j\in F_{c}}{\tilde{A}}_{j,c}}$
Reduced-form policy score (S_i,c_)	The combined evaluative score of option *i* in context *c*, defined as: *S*_*i*,*c*_ = *V*_*i*,*c*_ + *P*_*i*,*c*_. This approximates the negative of relevant expected free energy contributions (specifically expected risk and ambiguity) so that higher values indicate greater expected preference fulfillment and lower execution uncertainty. A fuller active inference model would decompose *S*_*i,c*_ into finer-grained components including expected outcomes, prior preferences, ambiguity, transition beliefs, and epistemic value.
Affordance field (F_c_)	The feasible set of food-related options available and achievable for an individual in a given context; in active inference terms, the feasible policy set.
Active inference	A framework in which agents select actions, or policies, that reduce expected uncertainty and risk relative to prior preferences, often described as minimizing expected free energy.
Pragmatic value (V_i,c_)	The expected alignment between the outcomes of option *i* in context *c* and the agent’s prior preferences - that is, how well the option is expected to deliver states the agent is organized to seek, such as satiety, solvency, social acceptance, health, or sustainability. In active inference terms, this is closest to the negative risk component of expected free energy: the expected alignment between predicted outcomes and preferred outcomes under a given policy. Pragmatic value is determined by the agent’s own preference structure; societal goals such as health or sustainability contribute to pragmatic value only to the extent that the agent has internalized them as prior preferences. Pragmatic value does not capture the habitual or cue-driven pull of familiar options, which is represented separately by the policy prior *H*_*i,c*_.
Policy prior (H_i,c_)	The learned tendency to select policy *i* in context *c*, accumulated through past selection history. In active inference, policy priors weight policy selection independently of the current expected free energy evaluation; in probability space, this prior can be represented as a multiplicative weighting on policy selection. Policies with strong priors are therefore more likely to be selected when alternatives score similarly on value and reliability. The policy prior is the primary formal mechanism used here to represent habitual, cue-driven, “Type 1” style behavior: familiar options feel immediately actionable because their selection pathway has been reinforced through repetition, not because they are freshly evaluated as optimal each time.
Policy-selection precision (γ)	A parameter controlling how deterministically the agent selects the highest-scoring policy in context *c*. High γ_*c*_ means the agent sharply favors the best-scoring option; low γ_*c*_ means policy selection is more diffuse, stochastic, or exploratory. Unlike an option-specific policy prior, γ_*c*_ is not specific to any one option: it governs the overall sensitivity of choice to differences between policies within a context, rather than the advantage of any particular option.
Normative value (N_i_)	The value of option *i* judged against public goals such as health, sustainability, nutritional adequacy, or reduced environmental impact. Normative value is assessed from an institutional or societal perspective and may diverge from pragmatic value: an option can have high normative value but low pragmatic value for a given agent if it does not align with their current preferences or does so only weakly relative to competing priorities such as cost, satiety, or convenience. Normative value is not a term within the active inference formalism, which models behavior from the agent’s perspective. It is introduced here as a policy construct to capture what institutional actors, such as governments, public health bodies, and food-system designers, want agents to prefer.
Predictive precision (P_i,c_)	The overall predictability with which option *i* can be enacted in context *c*, reflecting the inverse of the combined uncertainty associated with executing that policy, including outcome ambiguity, transition reliability, price volatility, preparation-time variance, spoilage risk, and household acceptance risk. Low *P*_*i,c*_ reflects volatile or failure-prone execution. In this paper, *P*_*i,c*_ refers to option-specific enactment precision and should not be confused with the policy-selection precision parameter γ_*c*_.
Epistemic value (ϵ_i,c_)	The expected information gain from selecting option *i* in context *c*: how much the agent would learn about uncertain states by trying this policy. Epistemic value is typically highest for novel or unfamiliar options and decays as the option becomes familiar and outcomes become predictable. It drives exploratory behavior, but only when the agent can afford the risk of uncertain outcomes. Epistemic value is part of the broader expected free energy framework but is not included in the reduced-form affordance equation used here.
Policy (active inference)	A candidate course of action, such as “cook from scratch”, “buy ready meals”, or “order takeaway”, evaluated in terms of expected outcomes, uncertainty, and risk. This is distinct from governmental policy.
Generative model	The agent’s learned internal model of how the world works: how actions lead to outcomes in specific environments. This model encodes both beliefs, meaning probabilistic expectations about what will happen, and prior preferences, meaning what counts as a good or bad outcome. In dietary choice, the generative model represents learned expectations about how food-related actions, such as cooking, ordering, and shopping, produce outcomes such as satiety, cost, time use, and household acceptance.
Beliefs	Probabilistic expectations about states of the world and likely outcomes of actions, such as “the store usually has stock”, “this meal will take 45 minutes”, or “my family will accept this”. Beliefs are updated through Bayesian inference when predictions are violated. For example, repeated stock-outs update the belief that sustainable options are reliably available at a given store.
Prior preferences	Desired states encoded in the agent’s model of the world, such as being healthy, avoiding hunger, staying within budget, minimizing waste, or eating sustainably. They define what counts as a good or bad outcome and shape the evaluation of different policies. While preferences can be learned or modified through cultural transmission and experience, such as acquiring sustainability concerns through education, they typically update more slowly than beliefs and through different mechanisms than simple evidence accumulation.
Expected free energy (information theory)	A quantity used in active inference to evaluate candidate policies in terms of anticipated risk, ambiguity, and epistemic value or expected information gain. In active inference, agents are modeled as selecting policies that minimize expected free energy. Note: despite the term “energy”, this is an information-theoretic quantity representing uncertainty and risk; it is distinct from thermodynamic or metabolic energy, such as kilocalories.
Leverage point	A place in a system where a small change produces a large shift; here, changes that alter what food options are feasible or reliably doable, such as price, access, infrastructure, retail cues, and social protection.

This is not a new observation in broad terms. The social and environmental determinants of behavior have been examined for decades. But much of the existing literature has remained at the level of association, for example, cataloging that poverty is associated with poorer dietary choices, without specifying the mechanisms through which these conditions shape which actions people actually take. Despite widespread recognition that information and labeling campaigns are insufficient (Vargas et al., 2025), the alternative - a “systems approach” - has largely remained a descriptive aspiration rather than a framework that specifies what to change and why it should work. This paper addresses that gap. It moves from association toward mechanism, de-stigmatizing dietary behavior by reframing the consumption of energy-dense foods not as a failure of self-regulation, but as a predictable response to structurally misaligned environments. It makes three related claims. First, the intention–behavior gap in sustainable eating is often better understood as a problem of feasible action than as a simple deficit of motivation. Second, the concept of dietary affordances captures this feasibility by focusing on how environmental constraints (e.g., time, funds, access, reliability) are translated into specific dietary choices, determining which actions are realistically achievable for particular people in particular situations. Third, active inference provides a formally grounded account of why dietary options that are more reliable, lower risk, and easier to execute, come to dominate eating behavior i.e., not because individuals lack motivation, but because the brain’s drive to minimize uncertainty favors policies that predictably work. By demonstrating that unsustainable dietary behavior is computationally rational given the environments in which it occurs, this framework shifts the locus of responsibility from individual consumers to the institutions that design and govern food systems.

Building on this argument, this perspective aims to (1) introduce the construct of dietary affordances as a formally grounded framework for understanding how food environments shape which dietary policies are realistically executable for particular people in particular contexts, (2) formalize this through a reduced-form model (derived from the active inference policy selection mechanism) that generates specific, testable predictions about the conditions under which sustainable options remain competitive or collapse within the feasible policy set, and (3) identify concrete leverage points where targeted changes to the affordance field can produce disproportionate shifts in dietary behavior, moving the “systems approach” from a descriptive aspiration to a predictive tool for policy. By developing this framework, we argue that closing the sustainable eating intention–behavior gap will depend less on further strengthening individual motivation and more on reshaping food environments so that the actions people can most safely and reliably enact are also those that support sustainable, nutritionally adequate diets.

## The intention–behavior gap

The intention–behavior gap is usually discussed through the lens of the Theory of Planned Behavior [TPB (Ajzen, 1991)]. In TPB, intentions are treated as the proximal determinant of behavior, shaped by attitudes, perceived social norms, and perceived behavioral control (PBC) - the sense that a behavior is realistically within one’s reach (Ajzen, 1991; Conner and Norman, 2022). On this account, stronger intentions and higher PBC should, in principle, translate into action. In practice, they often do not. Meta-analytic reviews show that TPB variables account for substantially more variance in intentions than in behavior: attitudes, norms, and PBC together explain around 40% of the variance in intentions, but only about 20%–30% of the variance in actual behavior (Armitage and Conner, 2001; Sheeran, 2002; Conner and Norman, 2022). In other words, strengthening intentions and perceived control improves prediction, but leaves most of what people do unexplained. Sustainable eating is no exception. Blanke et al. (2022) reported that, in a staff sample, intentions and self-reported behavior were both higher for healthy than for sustainable grocery shopping, but that a significant intention–action gap existed for both and was larger for sustainability. This suggests that, even when people endorse health and sustainability goals, sustainable options are experienced as harder to translate into everyday shopping practices. O’Keefe et al. (2016) similarly reported that consumers were willing, in principle, to reduce meat intake by around 20%, yet this willingness did not extend to broader, coordinated changes across the rest of the diet. More generally, many households maintain a strong reliance on convenient, processed foods even when they endorse health and sustainability goals (Blanke et al., 2022; Benton and Young, 2024). From an active inference perspective, this kind of reliance on convenient, processed foods can be understood as the selection of a policy that combines high predictive precision (reliable delivery of satiety under time and budget constraints), with a strong policy prior built through years of repeated successful enactment. Together, these give the processed option a high relative affordance weight, making it more likely to be selected than newer sustainable alternatives that lack both enactment reliability and accumulated selection history. This is consistent with work on grounded and situated cognition, which shows that specific food cues and contexts trigger rich simulations of consumption, making some options feel immediately desirable and ready to act on (Papies et al., 2020). In the present framework, this experiential readiness can be understood as the behavioral expression of a strong policy prior: options that have been repeatedly selected in a given context are not merely available but are experienced as the most obvious and actionable policies in that setting.

Conventional interpretations of the intention–behavior gap have largely emphasized individual-level explanations, that is limited self-regulation, weak motivation, competing goals, or biased risk appraisals (Sheeran, 2002; Conner and Norman, 2022). In that framing, the environment typically appears only as perceived barriers or facilitators, captured in measures of perceived behavioral control, and shortfalls between intentions and action are usually cast as individual failures to overcome such barriers rather than as properties of the wider system. Broader frameworks [including COM-B (Michie et al., 2011), socio-ecological models (Sallis and Owen, 2015), social practice theory (Shove et al., 2012), and choice architecture (Thaler et al., 2013)] have positioned environmental factors more centrally, but none provides a formally specified selection mechanism linking environmental properties to action selection at the point of choice (see Supplementary Material 1 for a detailed comparison).

In the systems-oriented account we develop here, informed by ecological psychology and active inference (Friston et al., 2012, 2013), we start from a different premise. We treat the gap as a structural misalignment between what people would like to do and the set of food-related actions that are realistically available, affordable, and low-risk in their everyday environments. By structural misalignment we mean that intentions point in one direction (e.g., “buy more sustainable foods,” “reduce meat consumption”), while the lowest-effort, lowest-risk, most predictable, and most habitual options in each context point somewhere else. This is evident, for example, in the difficulties participants report in enacting sustainable purchasing relative to health goals (Blanke et al., 2022), and in the selective application of sustainability intentions, such as willingness to reduce meat intake without broader changes to processed food consumption (O’Keefe et al., 2016). Put differently, many apparent “failures” of sustainable eating intentions are predictable responses to constrained dietary affordances: that is, when the everyday affordance field makes less sustainable options the safest, most habitual, and least disruptive policies, it is unsurprising that people choose them. This shift, from locating the problem primarily in individual shortcomings to focusing on the relational properties of people in environments, sets the stage for our use of “dietary affordances” as a more informative unit of analysis for understanding, and ultimately reducing, the sustainable eating intention–behavior gap. Before introducing dietary affordances in detail, it is useful to clarify what we mean by “intention” within this broader systems-oriented account.

### What are intentions?

Intentions occupy a central place in psychological and behavioral theories: they are typically treated as the immediate precursors to action and as key outputs of deliberation and decision-making. At a basic level, an intention is a commitment or plan to engage in a specific behavior in the future (e.g., “cook more plant-based meals on weeknights”). However, the nature of intentions, that is how they are formed, represented, and enacted, varies across theoretical frameworks.

A useful starting point is the distinction between different kinds of cognitive process. Dual-process accounts describe Type 1 processes as fast, automatic, and often unconscious, and Type 2 processes as slower, deliberative, and goal-directed (Kahneman, 2011; Evans and Stanovich, 2013). Related work on construal level theory distinguishes between high-level, abstract “why” representations and low-level, concrete “how” representations of action (Trope and Liberman, 2010). Some intentions are clearly the product of explicit, Type 2 reasoning (for example, deciding to follow dietary guidelines for health or environmental reasons) (Romijn et al., 2023), whereas others are more implicit, arising from repeated patterns of behavior and context that function as “automatic intentions” or habits (Wood and Rünger, 2016). In everyday life, expressed intentions often reflect a mixture of these elements: people articulate deliberate goals, but their actions are also guided by well-learned, context-sensitive response tendencies that require little conscious reflection.

In the active inference perspective we draw on, these different forms of intention can be viewed as expressions of prior preferences at different levels of abstraction (Friston et al., 2012, 2013; Pezzulo et al., 2018). High-level, verbally endorsed goals (for example, “eat more sustainably”) shape what we will later formalize as pragmatic value *V*_*i,c*_, that is the degree to which a policy’s expected outcomes align with the agent’s preferences. Low-level, habitual policies, by contrast, have acquired strong policy priors *H*_*i,c*_ through repeated successful selection in particular environments: they are enacted not because they are freshly evaluated as optimal each time, but because the selection pathway itself has been reinforced through repetition. On this view, intentions are not only what people say they want to do, but also the entrenched patterns of action that their nervous system has learned to default to under familiar conditions. This framing helps to bridge dual-process accounts and the systems-oriented analysis that follows, in which we focus on the dietary affordances that shape which policies are even available for those intentions to be expressed. It also clarifies why strengthening verbally stated intentions alone (e.g., through education campaigns) is unlikely to close the sustainable eating intention–behavior gap: increasing *V*_*i,c*_ for sustainable options does not weaken the policy prior *H*_*i,c*_ of the incumbent habit, nor does it increase the predictive precision *P*_*i,c*_ with which the sustainable action can be reliably executed. High-level goals must therefore compete with habitual policies whose selection is driven by strong policy priors, high enactment reliability, and tuning to the constraints of everyday life. In the next section, we therefore shift attention from intentions themselves to the affordance structure of food environments and ask how changing that structure can realign habitual actions and policies with people’s stated health and sustainability goals.

## Introducing dietary affordances

Intentions do not operate in a vacuum; they must be enacted within the hard constraints of reality. It is here that we introduce the construct of dietary affordances to occupy the crucial middle ground between internal intentions and observable behavior.

**FIGURE 1:**
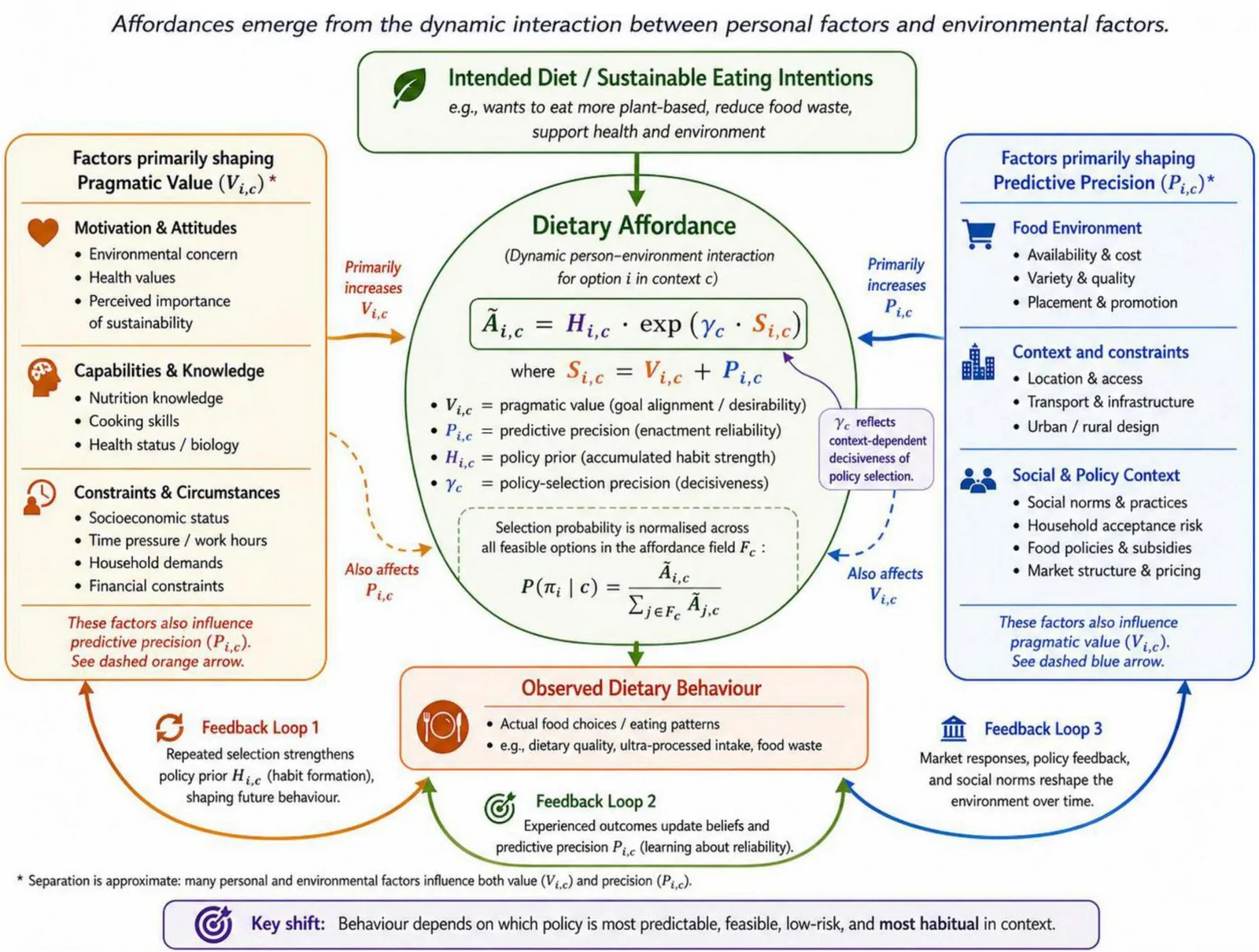
Dietary affordance as a dynamic person–environment system. The figure summarizes the components of the relative affordance weight for option i in context c : Ã_i,c_ = H_i,c_⋅exp(γ_c_⋅S_i,c_) where S_i,c_ = V_i,c_ + P_i,c_ is the reduced-form policy score. Each feasible option in the affordance field F_c_ has an associated relative affordance weight. **(Left)** Personal factors, such as preferences, capabilities, and time constraints, shape the option’s pragmatic value V_i,c_. **(Right)** Environmental factors, such as availability, cost, and spatial layout, shape the option’s predictive precision P_i,c_ by determining how reliably the option can be enacted in context. **(Center)** Value and precision contribute additively to the policy score S_i,c_, which is transformed non-linearly through exponentiation and weighted by the policy prior H_i,c_, representing accumulated habit strength. The parameter γ_c_ reflects the context-dependent decisiveness of policy selection. Selection probability is determined by normalizing across the feasible affordance field, so that affordance strength is relative to competing options. Bottom: three feedback loops capture the dynamic properties of the system. Loop 1, repeated selection strengthens the policy prior H_i,c_, increasing habitual selection of that option; Loop 2, experienced outcomes update beliefs and predictive precision P_i,c_, reflecting learning about reliability; Loop 3, market responses, policy feedback, and social norms reshape environmental conditions over time. Dashed arrows indicate that the separation between value and precision is approximate: many personal and environmental factors influence both dimensions.

Drawing on ecological psychology, we define affordances not merely as physical properties, but as relational possibilities for action that emerge from the structural coupling between an environment and an individual (Gibson, 1977; Figure 1). A setting does not afford the same actions to everyone; its opportunities are dictated by the specific capabilities, resources, and needs of the person who encounters it (Lobo et al., 2018; Papies et al., 2020). The same supermarket offers a vastly different “menu” of choices to a wealthy retiree than it does to a time-poor single parent.

Crucially, from an active inference perspective, these affordances define the practical horizon of action sequences an agent can realistically consider (Friston et al., 2012, 2013, 2016). This reframes the “choice” to eat unsustainably. For a parent working irregular hours with limited funds, “heat a ready meal” or “order takeout” are not failures of self-regulation; they are reliable, low-risk solutions to the urgent problem of feeding a household and, through repetition, can become entrenched habitual defaults. In such a context, “prepare a plant-based meal from scratch” may not disappear absolutely, but its probability of selection can become vanishingly small: it may be too risky, too time-consuming, or too financially precarious to function as a viable option. By contrast, a household with stable income and a well-equipped kitchen inhabits a different affordance field, where batch cooking and experimenting with legumes may become realistic, low-risk options.

More formally, we define dietary affordances as the set of food-related actions that are realistically available and achievable for an individual in a given context. These affordances emerge from the dynamic interaction between personal factors, such as income, time, cooking skills, health status, and preferences, and environmental factors, such as price, retail layout, transport, cooking facilities, and social norms. When these factors align, the environment can act as a scaffold that transforms a weak preference into an entrenched habit through reliable outcomes and repeated selection.

In the present framework, this non-linearity is central to the affordance concept: options do not become behaviorally dominant simply because they are valued, but because value, reliability, and selection history combine to determine which policies are enacted. Drawing on active inference, we formalize this through a reduced-form approximation of policy selection (Box 1). The notation is intended to make the framework’s assumptions explicit while retaining an empirically tractable form. In active inference, agents select policies by minimizing expected free energy, which is commonly decomposed into pragmatic components such as expected risk and ambiguity, and epistemic components such as expected information gain. Policies with lower expected free energy are more likely to be selected, with the determinism of this selection governed by a softmax function (Friston et al., 2016, 2017; Parr and Friston, 2019). In addition, agents carry learned policy priors (accumulated tendencies to select particular courses of action) that weight policy selection independently of the current expected free energy evaluation (Friston et al., 2013).

In the present framework, we approximate this structure as follows. For each feasible dietary option *i* in context *c*, let *V*_*i,c*_ denote pragmatic value (the expected alignment between the option’s outcomes and the agent’s prior preferences); and *P*_*i,c*_ denote predictive precision (the inverse of the combined uncertainty associated with executing the policy, including outcome ambiguity, transition reliability, price volatility, preparation-time variance, spoilage risk, and household acceptance risk). We combine these into a reduced-form policy score:


$$S_{i,c}=V_{i,c}+P_{i,c}$$

where higher values indicate greater expected preference fulfillment and lower execution uncertainty. *S*_*i,c*_ approximates the negative of relevant expected free energy contributions from risk and ambiguity. A fuller active inference model would decompose *S*_*i,c*_ into finer-grained components including expected outcomes, prior preferences, ambiguity, transition beliefs, and epistemic value. The relative affordance weight is then:


$${\tilde{A}}_{i,c}=H_{i,c}\cdot \text{exp}\,\left(\gamma_{c}\cdot S_{i,c}\right)$$

where *H*_*i,c*_ denotes the policy prior (the learned tendency to select this option in this context, accumulated through past selection history); and γ_*c*_ denotes policy-selection precision (the degree to which the agent deterministically favors the highest-scoring policy in context). Epistemic value is not included in this reduced-form expression because the present section focuses on familiar, repeated dietary choices; we return to epistemic value when discussing the initiation problem below.

The probability of selecting option *i* is obtained by normalizing across the feasible affordance field *F_c_*:


$$P\,\left(\pi_{i}|c\right)=\frac{{\tilde{A}}_{i,c}}{\sum_{j\in F_{c}}{\tilde{A}}_{j,c}}$$

Affordance strength is therefore relative: an option’s behavioral pull depends not on its score in isolation, but on how it compares to everything else available in context. This is consistent with ecological accounts in which affordances are relational and context-dependent rather than fixed properties of objects (Gibson, 1977; Rietveld and Kiverstein, 2014).

Several features of this formulation are important. First, pragmatic value and predictive precision contribute additively inside the exponent, reflecting the additive structure of the reduced-form policy score and loosely corresponding to expected preference fulfillment and reduced ambiguity in expected free energy. The non-linearity that produces threshold-like effects arises not from multiplication between value and precision, but from the exponential transformation and normalization across competing policies. Because policy selection operates through a softmax, even modest reductions in predictive precision can produce sharp drops in selection probability, particularly when competing options score well. This means that when *P*_*i,c*_ is very low (because outcomes are volatile or the option is complex and failure-prone) the option’s relative affordance weight can collapse even when the individual values the goal, especially when competing options have higher reliability or stronger policy priors.

Second, the policy prior *H*_*i,c*_ enters multiplicatively as a prior weighting on the unnormalized affordance weight, consistent with the role of priors in Bayesian policy selection (Friston et al., 2013). A ready meal that has been selected repeatedly under time pressure may acquire a strong policy prior: it is more likely to be selected not because the agent freshly evaluates it as optimal each time, but because the selection pathway itself has been reinforced. This is the primary mechanism through which habitual, automatic behavior emerges: familiar options feel immediately actionable because their prior is high, not because they are continuously re-evaluated against alternatives.

Third, the exponential structure generates the empirically distinguishable predictions that motivate the framework. It predicts non-linear threshold-like effects, such that small reductions in reliability can produce abrupt drops in sustainable food selection once household viability thresholds are crossed (Table 1; H1). It also predicts Constraint × Reliability interactions, such that low income, food insecurity, or time pressure should amplify the behavioral impact of price volatility, stock-outs, preparation-time variance, or household rejection risk, because constrained agents may be operating in regions of the policy-selection function where small reductions in the policy score produce large changes in selection probability (Table 1; H2). These predictions can be tested by comparing linear models with models that include interaction terms or non-linear threshold parameters.

**TABLE 1:** Testable hypotheses derived from the dietary affordance framework.

Cluster	Hypothesis	Prediction	Example test/operationalization
Core mechanism	H1. Threshold effects	Dietary quality should not degrade only linearly with income. Instead, small changes in price, spoilage risk, or failure costs should produce sudden behavioral shifts once costs cross household viability thresholds.	Use regression discontinuity, piecewise regression, splines, or change-point analysis to test for non-linear drops in sustainable food consumption at income, budget, or resource thresholds. Track dietary quality across income deciles and look for discontinuities rather than smooth slopes.
Core mechanism	H2. Non-linear precision penalty	Because policy selection operates through a non-linear (exponential/softmax) transformation, sustainable options should show steeper consumption drops under constraint than predicted by linear models where constraint affects value and precision independently	Use panel data to regress dietary choices on income, price variance, and their interaction. A significant Constraint × Price Volatility interaction should predict sustainable food consumption above and beyond the main effects of income and price variance.
Intervention mechanism	H3. Information affects value, not precision	Eco-labeling or information campaigns should increase stated concern, intentions, or perceived value without changing behavior when environmental reliability remains low.	Use a randomized trial of an information intervention measuring intentions, behavior, and environmental precision proxies such as stock-outs and price volatility. Mediation analysis should show information → intentions, but a weak intention → behavior path when precision is low.
Intervention mechanism	H4. Intention × Precision interaction	Intentions should predict behavior most strongly when objective precision is high, such as stable prices, reliable stock, and low failure cost. This interaction should remain after controlling for perceived behavioral control.	Use hierarchical regression: behavior predicted by intention, perceived behavioral control, environmental precision, and Intention × Precision. Environmental precision can be measured using price coefficient of variation, stock-out frequency, and preparation-time variance.
Intervention mechanism	H5. Race against precision	First encounters with sustainable options should shape long-term adoption more strongly than later exposures. Products introduced with low or mediocre precision may acquire low expected reliability that resists subsequent marketing or product improvement.	Use longitudinal cohort or product-adoption data tracking initial versus later exposures to new sustainable products. Early failure, such as waste, rejection, time overrun, or unexpected cost, should predict long-term non-adoption even after later improvements.
Intervention mechanism	H6. Epistemic value down-weighting under constraint	Exploration of novel sustainable options should decrease as household constraint increases, even when stated sustainability values remain high.	Use purchase variety or novelty measures regressed on constraint indicators. Transaction data could measure the number of unique new sustainable products tried per quarter, stratified by income, food insecurity, or time-pressure proxies.
Policy leverage point	H7. Precision substitution	Carbon taxes on meat, or similar fiscal restrictions, without viable high-precision sustainable alternatives may shift consumption toward other high-precision options, such as ultra-processed ready meals or processed plant-based products, rather than fresh vegetables.	Use a natural experiment or quasi-experimental design around tax introduction. Household panel data pre- and post-policy can track category substitution patterns: meat to fresh produce versus meat to ultra-processed or other high-reliability substitutes.
Policy leverage point	H8. Subsidize reliability > subsidize health	Subsidies for frozen, tinned, or otherwise reliable sustainable options should produce larger and more sustained behavioral shifts than equivalent subsidies for fresh produce alone, where spoilage risk and preparation burden remain high.	Use a field experiment or voucher trial varying subsidy targets. Compare uptake, redemption, repeat purchase, waste, and sustained consumption for fresh-produce subsidies versus shelf-stable or low-preparation sustainable subsidies.
Policy leverage point	H9. Operational consistency > physical proximity	Dietary quality should correlate more strongly with retailer operational reliability, such as consistent opening hours, stock availability, and service predictability, than with distance to healthy food outlets alone.	Combine geographic distance measures with retailer reliability scores, including opening-hour variance, stock-out frequency, price stability, and audit or review data. Test whether reliability predicts consumption after controlling for physical proximity.
Policy leverage point	H10. Policy prior dominance	Repeat-purchase history for a dietary option should predict future selection independently of stated value and objective environmental precision. Interventions that increase value or precision for sustainable options should be less effective when the incumbent unsustainable option has a strong policy prior, and more effective when the incumbent prior has been destabilized (e.g., through product discontinuation, relocation, or routine disruption).	Use transaction panel data to test whether repeat-purchase frequency predicts future selection after controlling for stated preference and environmental precision proxies. Test whether intervention effectiveness (e.g., a subsidy or default swap) is moderated by prior purchase frequency of the incumbent option. Life transitions such as moving house, changing jobs, or retirement can serve as natural experiments in which existing policy priors are weakened, predicting a window of greater susceptibility to precision-based interventions.

To illustrate, consider a workplace cafeteria. An office worker who intends to eat more healthily is more likely to do so when the cafeteria reliably affords that behavior (through a visible, appealing salad bar with consistent stock and predictable pricing) than when only energy-dense options are prominently displayed. In the present framework, the reliable cafeteria increases *P*_*i,c*_ for the healthy option and, if the worker has selected it before, can also strengthen *H*_*i,c*_. Both contribute to a higher relative affordance weight. Crucially, the worker who has repeatedly chosen the salad bar may build a policy prior that makes the selection increasingly automatic: the healthy option no longer requires deliberative comparison against alternatives each time but is enacted as a habitual default.

Each afforded policy carries expected costs, risks, and rewards, including the probability of food waste, the impact on the weekly budget, and the social acceptability of the meal. Policies that repeatedly work under these constraints acquire both higher predictive precision and stronger policy priors, making them increasingly likely to be selected and increasingly resistant to displacement by novel alternatives. This formulation allows us to move beyond treating the “systems approach” as a descriptive metaphor. By offering a formally grounded approximation linking environmental reliability and habit formation to action selection, it provides a mechanistic account of why small structural changes, or leverage points, may produce disproportionate shifts in dietary behavior.

The present equation should be understood as a reduced-form approximation rather than a full Bayesian generative model. In a fully specified active inference model, each dietary option would be represented as a policy π_*i*_, a sequence of actions evaluated under a generative model of the agent-in-context, with policy selection modeled as a softmax function over expected free energy (Friston et al., 2016, 2017). The policy score *S*_*i,c*_ functions as a positive reduced-form approximation of the negative of expected free energy contributions from risk and ambiguity, where higher values indicate greater expected preference fulfillment and lower uncertainty. The purpose of this approximation is to make the framework empirically accessible, allowing researchers to test predictions using observable environmental variables such as price volatility, stock-out rates, preparation-time variance, and household rejection risk, without first estimating latent-state posteriors, transition probabilities, or full generative-model parameters. The later section, “Operationalizing the Framework: From Theory to Testable Predictions,” specifies how these variables can be measured and operationalized. Table 1 then translates the framework into a set of testable hypotheses, including threshold effects, non-linear precision penalties, Intention × Precision interactions, and precision substitution. Together, these sections clarify that the present framework is not intended as a completed computational model, but as an empirical scaffold for testing when and why sustainable options remain competitive or collapse within the feasible policy set.

Having established the affordance framework, we now apply it to reinterpret a central question in behavior-change research: why do automatic, habitual policies so often dominate reflective intentions, particularly under constraint?

## Explaining the gap: why unsustainable policies dominate

**FIGURE 2:**
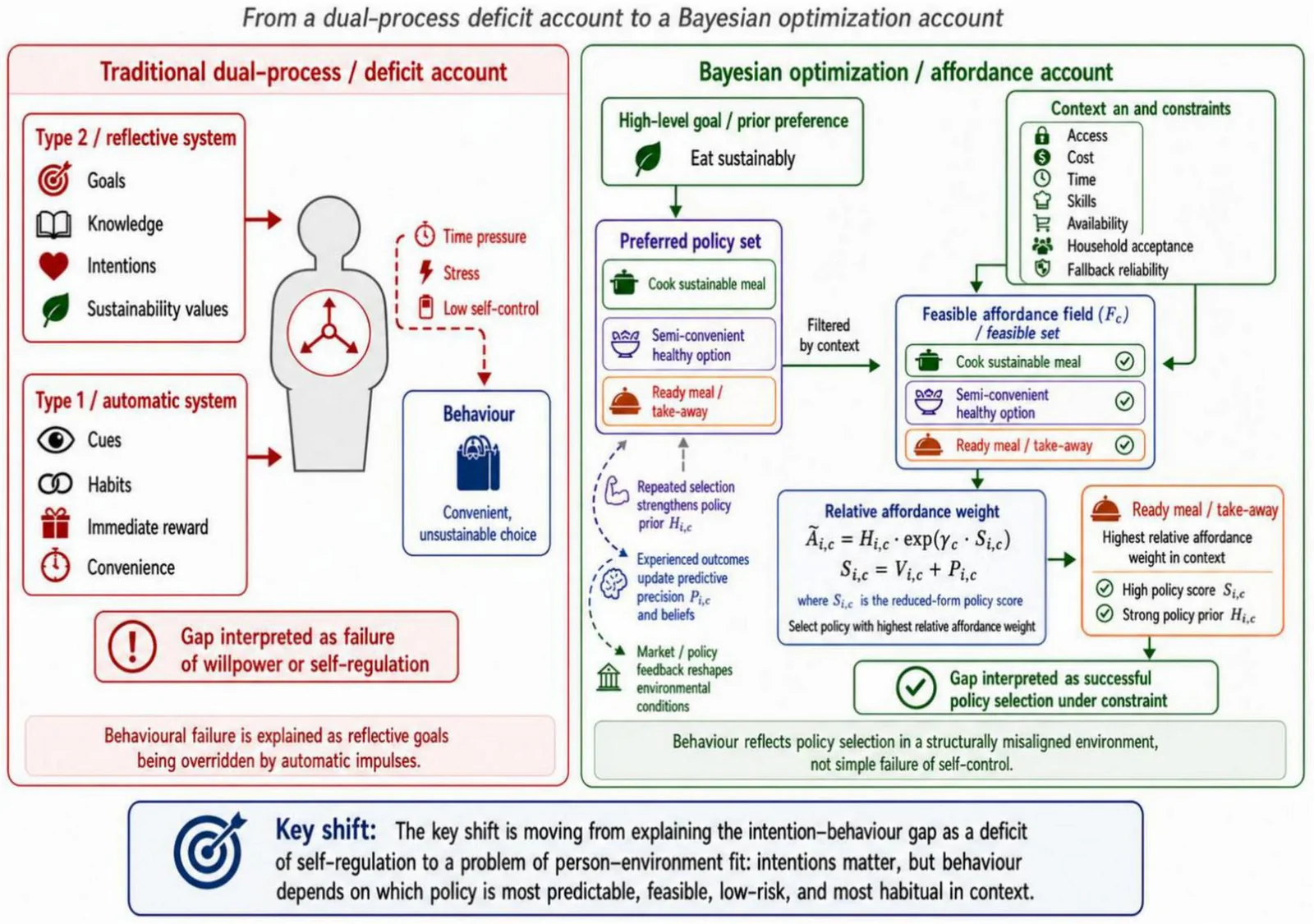
Reframing the intention–behavior gap. This figure contrasts two ways of understanding the intention–behavior gap in sustainable eating. The left panel presents a traditional dual-process or deficit account, in which behavior is interpreted as the outcome of conflict between reflective Type 2 processes (goals, knowledge, intentions, sustainability values) and automatic Type 1 processes (cues, habits, immediate rewards, convenience). Contextual pressures such as time pressure, stress, and low self-control are treated as factors that undermine reflective control, leading to convenient, unsustainable choices. The gap is therefore interpreted primarily as a failure of willpower or self-regulation. The right panel presents the dietary affordance account. Here, behavior is understood as policy selection under constraint. A high-level goal or prior preference (for example, “eat sustainably”) generates a preferred policy set, but behavior depends on the subset of options that are feasible in context — the affordance field *F_c_*. This feasible set is shaped by contextual constraints that determine predictive precision, including access, cost, time, skills, availability, household acceptance, and fallback reliability. Within this set, policy selection reflects the relative affordance weight Ã_*i*,*c*_ = *H*_*i*,*c*_⋅exp(γ_*c*_⋅*S*_*i*,*c*_) where: *S*_*i*,*c*_ = *V*_*i*,*c*_ + *P*_*i*,*c*_ is the reduced-form policy score combining pragmatic value and predictive precision, *H*_*i,c*_ denotes the policy prior, and γ_*c*_ denotes policy-selection precision. The intention–behavior gap emerges when the preferred policy set exceeds the feasible policy set. Under constraint, the ready meal or takeaway may have the highest relative affordance weight — not only because of a high policy score *S*_*i,c*_ but also because of a strong policy prior *H*_*i,c*_ built through repeated selection. The feedback pathways capture how repeated selection strengthens the policy prior, experienced outcomes update predictive precision and beliefs, and market or policy feedback reshapes environmental conditions. On this account, the intention–behavior gap is not a failure of self-control but a predictable outcome of policy selection in a structurally misaligned environment.

Having reframed the intention–behavior gap as a structural misalignment rather than a self-regulatory deficit, we can now examine what specifically gives unsustainable options their behavioral advantage. In constrained food environments, processed and ultra-processed foods score well on every dimension of the selection equation. They offer predictable satiety, fixed prices, and minimal preparation risk, all contributing to high predictive precision *P*_*i,c*_ (Friston et al., 2017; Parr and Friston, 2019). Crucially, this predictability extends to the sensory experience itself (Friston, 2010; Friston et al., 2017). While natural foods vary in quality (a ripe vs. unripe fruit), ultra-processed foods are engineered for hyper-consistency, delivering predictable sensory outcomes every time (Friston et al., 2012; FitzGerald et al., 2015). In active inference terms, this lack of sensory variance reduces outcome ambiguity, contributing to lower expected free energy for the processed option (Friston, 2010; Friston et al., 2012, 2015). Meanwhile, repeated selection under time and budget pressure strengthens the policy prior *H*_*i,c*_, making the processed option increasingly automatic and resistant to displacement. Sustainable alternatives, by contrast, may carry volatile pricing, uncertain preparation demands, spoilage risk, and weak or absent policy priors. When these factors combine under pressure, the system does not “fail” by choosing the processed option; it settles on the policy that most reliably minimizes expected risk in context (Figure 2).

This analysis exposes a specific limitation of interventions that focus on manipulating representations while leaving the material environment unchanged. Experimental paradigms often rely on altering psychological distance or construal level while holding the practical choice context constant (Trope and Liberman, 2010). Such manipulations can increase pragmatic value *V*_*i,c*_ by making sustainability a more strongly weighted preferred outcome. However, they typically do not increase predictive precision *P*_*i,c*_ i.e., the reliability with which the relevant actions can be executed in context, nor do they weaken the policy prior *H*_*i,c*_ of the incumbent habitual option. If the environment remains volatile or high-friction, increasing goal salience risks creating a mismatch: the person values the sustainable outcome more, reflected in higher *V*_*i,c*_, but their generative model still predicts that the sustainable action is likely to fail, reflected in low *P*_*i,c*_, and the habitual default retains a strong prior built through years of successful selection. Once the artificial support of the intervention is removed, the system predictably reverts to the policy with the strongest combination of reliability and habit strength. Closing the gap therefore requires a shift from “motivating individuals” to “engineering reliability and building new habits”: reshaping the material, social, and institutional affordance field so that sustainable policies become the safest, most predictable route to satiety, while supporting enough successful repetition to establish competing policy priors.

## Aligning people and places: an active inference approach to environmental change

The previous section showed why unsustainable options dominate under constraint: they combine high predictive precision, strong policy priors, and predictable sensory outcomes in a way that sustainable alternatives typically cannot match. The question now is what can be done. In active inference, behavior is guided by a generative model that encodes both prior preferences (the states the agent is organized to seek, such as satiety, solvency, and health) and beliefs about how reliably different policies will realize those states in a given environment (Friston et al., 2012; Parr and Friston, 2019; Da Costa et al., 2020). Policies are evaluated in terms of expected free energy: the anticipated risk of failing to achieve preferred outcomes and the uncertainty associated with execution (Friston, 2010; FitzGerald et al., 2015; Friston et al., 2017). Crucially, this evaluation is context dependent. What counts as a feasible policy depends on the affordances the environment provides - the foods stocked, the prices set, the time available, the reliability of fallback options, and the social conditions under which meals are prepared and consumed (Gibson, 1977; Withagen et al., 2012; Rietveld and Kiverstein, 2014). The issue is therefore not necessarily that people lack sustainable preferences. Rather, they may believe - often accurately - that the sustainable policy is costly, unavailable, time-consuming, socially risky, or likely to fail in the current context.

This suggests two broad routes through which sustainable outcomes can be pursued. The first is individual action: the agent selects policies intended to realize preferred outcomes, such as planning meals, cooking from scratch, or comparing products. This is the route implicitly targeted by most behavior-change interventions. The second is niche construction: the environment is modified so that preferred outcomes become easier to realize i.e., through changes to pricing, availability, infrastructure, defaults, and social supports (Day et al., 2003; Laland and Sterelny, 2006; Constant et al., 2022).

By identifying the environment as a primary source of decision volatility, this framework exposes the limitations of relying primarily on the first route. Demanding that households under economic strain “care more” about sustainability is effectively asking them to select a low-precision policy: one that may carry high financial, temporal, or social risk without altering the underlying constraints (Feldman and Friston, 2010). It is neither ethically defensible nor behaviorally realistic to expect individuals to override high-precision priorities, such as solvency and satiety, with abstract, high-variance intentions when the environment offers no viable path to enact them (Friston et al., 2017; Parr and Friston, 2019). Instead, the goal is structural alignment: configuring food environments so that sustainable choices become high-precision policies with the opportunity to build competing policy priors through repeated successful enactment. The following sections examine first why the individual action route faces systematic limits, then how constrained environments amplify those limits, and finally where niche construction can intervene most effectively.

## Implications for intervention: value, timing, and the initiation–maintenance problem

In this section we highlight three implications for intervention design. First, information-based approaches may increase the perceived value, salience, or normative importance of sustainable eating, but they often do not increase the precision with which sustainable actions can be enacted. Second, timing matters because early experiences shape both predictive precision and policy priors: if sustainable options are introduced with high failure costs or unreliable execution, they fail to build the policy priors needed for habitual adoption, and the resulting weak priors resist later correction. Third, initiation and continuation are distinct intervention problems. Initial uptake requires safe exploration, so that people can try sustainable options without jeopardizing satiety, time, or budget. Sustained behavior change requires reliable enactment, so that repeated experience converts the option from a novel experiment into a low-risk default.

### Value, precision, and habit: why information is insufficient

Crucially, this framework explains the persistent underperformance of information-based campaigns (Kelly and Jewell, 2018). Traditional approaches assume that providing knowledge, for example through eco-labeling, updates the generative model and thereby changes behavior. However, within active inference, information may increase the pragmatic value *V*_*i,c*_ of a goal by making sustainability more desirable, while rarely increasing the predictive precision *P*_*i,c*_ of the actions required to achieve it. Moreover, information does nothing to weaken the policy prior *H*_*i,c*_ of the incumbent habitual option — the entrenched tendency to select familiar, reliable foods that has been built through years of successful repetition. If the sustainable action remains high-effort and uncertain, and the habitual default retains a strong prior, the system will continue to select the familiar policy even when the agent is well-informed. Knowledge is a weak force when pitted against the combined weight of low enactment reliability and strong habit strength. Effective policy must therefore reshape the affordance field *F_c_* through income supports, pricing, and infrastructure, so that the policies that are safest and most feasible for constrained households are also those that support sustainable diets, while also creating conditions for sustainable options to build their own policy priors through supported repetition.

### The temporal constraint: a race against precision

This framework introduces a critical temporal dimension to intervention design. In active inference, repeated experience shapes behavior through two related but distinct mechanisms. First, the agent’s beliefs about the outcomes of a policy become more precise: outcome distributions tighten as the agent learns what to expect (Vossel et al., 2013). Second, and crucially, the policy prior H_i,c_ strengthens with repeated successful selection: the more often a policy is enacted in a context, the more likely it is to be selected again, partly independently of its current evaluative score (Friston et al., 2016). Together, these mechanisms mean that early experiences with a food option can “lock in” behavior, not only by shaping what the agent expects, but by reinforcing which policy the agent defaults to.

When a food environment or product is novel, both policy priors and outcome beliefs are relatively weak, and behavior is more sensitive to external cues such as eco-labels, price promotions, or trial offers. With repetition, policy priors strengthen and outcome beliefs tighten, making the policy increasingly habitual and resistant to new information, even when subsequent educational appeals change stated preferences.

This implies a race against precision for sustainable foods. Interventions must be deployed at the point of market entry, when affordance fields shift and existing priors are destabilized. If sustainable options are introduced with low predictive precision, for example plant-based alternatives that are expensive, unpredictable in texture, or hard to cook, consumers may fail to select them repeatedly enough to build a strong policy prior. A salient negative experience may therefore weaken future selection by lowering expected reliability and preventing the policy prior from strengthening, even if later versions of the product improve. A market illustration of the challenge of establishing viable plant-based defaults is the withdrawal of plant-based product lines by major manufacturers from some markets in recent years. Although such cases cannot establish the mechanism, they illustrate the commercial risk of introducing sustainable alternatives before they are sufficiently affordable, familiar, and reliable. The goal of niche construction, therefore, is not just to make sustainable options available, but to ensure that their initial affordance weight is robust enough — through adequate predictive precision and low-risk first encounters — to seed a policy prior before the window of plasticity closes.

### Epistemic value and the initiation problem

The temporal argument above also exposes a second barrier. Getting a person to try a sustainable option for the first time is often an exploration problem, not a persistence problem. In active inference, policy selection is guided by expected free energy, which combines a pragmatic drive to secure preferred outcomes by reducing expected risk and an epistemic drive to reduce uncertainty by seeking information that resolves ambiguity (Friston et al., 2015; Parr and Friston, 2019). This matters because novelty can be attractive in principle — trying an unfamiliar food could yield valuable information — but only when exploration is safe.

We can incorporate this directly into the dietary affordance account by reintroducing epistemic value and making explicit that all components of affordance weight evolve over time. For option *i* in context *c*, the relative affordance weight at time *t* can be expressed as:


$${\tilde{A}}_{i,c}\,\left(t\right)=H_{i,c}\,\left(t\right)\cdot \text{exp}\,\left[\gamma_{c}\,\left(S_{i,c}\,\left(t\right)+\epsilon_{i,c}\,\left(t\right)\right)\right]$$

where:


$$S_{i,c}\,\left(t\right)=V_{i,c}\,\left(t\right)+P_{i,c}\,\left(t\right)$$

is the reduced-form policy score as defined above, and ϵ_*i*,*c*_(*t*) captures epistemic value: the temporary information bonus of trying something uncertain in order to learn what happens, which is typically highest early in exposure when outcome beliefs are imprecise. *H*_*i*,*c*_(*t*) captures the policy prior: the accumulated tendency to select this option, which starts near-neutral for novel options and grows through repeated successful selection. For established options where epistemic value has decayed, this reduces to the base equation:


$${\tilde{A}}_{i,c}=H_{i,c}\cdot \text{exp}\,\left(\gamma_{c}\cdot S_{i,c}\right)$$

As the option is repeated, ϵ_*i*,*c*_(*t*) naturally decays because uncertainty is resolved: the agent has learned what the option delivers. The long-run fate of the behavior then depends on whether experience increases *P*_*i*,*c*_(*t*) and builds *H*_*i*,*c*_(*t*) enough for the relative affordance weight to remain competitive under real-world constraints, even after the epistemic bonus has faded (Friston et al., 2015; Parr and Friston, 2019).

This formulation sharpens the policy implication. Information-based campaigns implicitly assume that consumers will experiment with sustainable options and learn their value over time. Under constraint, however, the epistemic value of novelty may be outweighed by expected risk: when exploration could mean a wasted meal, a rejected dinner, or a budget overrun, the safer high-prior default remains more likely to be selected. Novel sustainable options are therefore less likely to be sampled or may be sampled once and abandoned after a single failure, with no stable policy prior built.

The design target is therefore twofold. Policy must first reduce the penalty for exploration so that initial sampling can occur without jeopardizing satiety, time, or budget, for example through subsidized trial portions, money-back guarantees, or low-commitment formats such as meal kits with familiar fallback options. It must then ensure reliable enactment, so that repeated experience increases *P*_*i*,*c*_(*t*) and builds *H*_*i*,*c*_(*t*), converting the option from a novel experiment into a habitual default (Friston et al., 2016).

## From theory to food systems: dietary affordances under food insecurity

The preceding sections established general principles: intervention must target precision and habit, not just value; timing matters because early experiences shape policy priors; and initiation and maintenance are distinct problems. We now apply this lens to households navigating food insecurity and cost-of-living pressures as a stress test of the framework - a context where the compression of the policy set is most visible and the consequences of misaligned intervention are most severe.

Food insecurity is not simply a condition of having fewer resources; it changes the decision problem itself. Food insecurity research has long recognized that insecurity is fundamentally a problem of uncertainty, that is not knowing whether adequate food will be available, affordable, acceptable, or sufficient when needed (Radimer et al., 1992; USDA Economic Research Service, 2026; Coates et al., 2006). The present framework builds on this insight by specifying the decision mechanism through which such uncertainty shapes behavior. When money, time, energy, and fallback options are scarce, the cost of uncertainty rises sharply: a rejected meal, spoiled ingredient, price increase, or failed recipe can mean wasted money, missed satiety, or no viable alternative. Socioeconomic conditions therefore do not merely limit what people can buy; they alter the feasible policy set by changing the expected risk, reliability, and failure cost of different dietary policies. When a household cannot be confident that a sustainable meal will deliver satiety, remain within budget, avoid waste, or be accepted, that meal loses predictive precision and may no longer function as a viable policy.

To understand this as a generative process, consider the affordance field of a household operating under strict budget and time constraints (Loopstra et al., 2015). For such a household, preferred outcomes (avoiding hunger, maintaining financial solvency, minimizing waste, and securing household acceptance) are highly consequential. The preference for health or sustainability may still be present, but it competes with immediate, high-precision priorities that carry severe consequences if violated and that have acquired strong policy priors through years of repeated successful selection. When this household evaluates the policy “buy fresh, sustainable ingredients,” the expected free energy may be high: the financial cost may be volatile relative to the remaining budget, preparation time may be uncertain, and the meal may fail through spoilage, cooking difficulty, or household rejection (Wrieden et al., 2019).

By contrast, the policy “buy a processed ready meal” may offer high enactment precision. The monetary cost is known at the point of purchase, the time and skill demands are minimal, and the sensory and satiety outcomes are predictable (Remnant and Adams, 2015; Hall et al., 2019). In active inference terms, when sustainable options carry greater financial, temporal, sensory, or social uncertainty, their relative affordance weight can collapse to the point where they cease to function as viable competitors, even when their normative value remains high. The system minimizes expected risk by selecting the policy most likely to secure satiety within the constraints of the current environment (FitzGerald et al., 2015; Young et al., 2021).

Recent data from the United Kingdom cost-of-living crisis illustrate this mechanism. Rising food and energy prices have compressed household policy sets by forcing trade-offs between housing, utilities, and food (Hill et al., 2023; Meadows et al., 2024). In a Welsh national survey, approximately 20% of respondents reported eating less food and 12% reported eating more processed foods specifically in response to rising costs (Hill et al., 2023). Crucially, this shift occurred despite high reported concern for health and sustainability (Culliford and Bradbury, 2020). This supports the active inference account: dietary behavior adapted not because values disappeared, but because the affordance field shifted, making energy-dense, processed options the policies most capable of reliably reducing the expected risk of hunger, waste, and financial overrun.

### Inequality and the compression of the policy set

The food insecurity analysis above illustrates a general principle: inequality reshapes not only what people can buy, but which dietary policies they can safely select. Standard accounts often assume that if a sustainable option is physically present (for example, stocked in a local shop) it is therefore “available” to the consumer. An active inference perspective challenges this assumption: an option is only functionally available if it can be executed with sufficient reliability, given real constraints on time, money, mobility, fatigue, and competing demands. In the present framework, the relative affordance weight of an option depends on pragmatic value *V*_*i,c*_, predictive precision *P*_*i,c*_, and the policy prior *H*_*i,c*_, with value and precision contributing additively to the policy score *S*_*i,c*_ that is then transformed non-linearly through the softmax. Because of this exponential structure, small reductions in reliability under constraint can produce sharp drops in selection probability, particularly when competing options (such as familiar processed foods with strong policy priors) score well.

The crucial point is that these reliability penalties are not evenly distributed. For a household with a financial buffer, a failed £3 ingredient purchase may be irritating but absorbable. For a household operating at the edge of solvency, the same failure may mean a missed meal or the loss of the only available dinner option. In affordance terms, the option’s predictive precision *P*_*i,c*_ collapses because the household can no longer absorb uncertainty, and the sustainable option’s relative affordance weight drops sharply — not gradually — even if its pragmatic or normative value remains unchanged. Meanwhile, the processed alternative retains both high predictive precision and a strong policy prior built through repeated successful selection, widening the gap between the two options’ selection probabilities. Food insecurity therefore changes not only what households can afford, but what they can risk.

This produces a specific, testable prediction. As household resources contract, dietary behavior should not deteriorate only gradually. Instead, small changes in price, spoilage risk, or available fallback options may produce sudden shifts once the cost of failure crosses a viability threshold (Table 1; H1). In this high-stakes context, decision-making narrows as scarcity taxes mental bandwidth and increases sensitivity to immediate risk (Mani et al., 2013). Ultra-processed foods are therefore not selected merely for “convenience”; they are selected because they are reliably executable. Costs are predictable, preparation demands are low, shelf-life is longer, and outcomes are consistent. Consistent with this account, food insecurity is associated with higher intake of ultra-processed foods, with proposed pathways including economic constraint, stress, and coping strategies that favor shelf-stable, ready-to-eat options (Leung et al., 2022).

Closing the sustainable eating intention–behavior gap for constrained households therefore cannot rely on increasing motivation alone. It requires redesigning food environments so that sustainable options carry lower failure costs, greater fallback security, and higher enactment precision - and then supporting enough successful repetition for these options to build the policy priors that make them habitual. Only then can sustainable policies remain within, and eventually dominate, the feasible policy set under real-world constraint.

## Bridging the intention–behavior gap: leverage points in the affordance field

The preceding sections established that the intention–behavior gap in sustainable eating is driven not by motivational deficit but by structural misalignment: information targets value but not precision or habit; early encounters shape policy priors in ways that resist later correction; and constrained households face amplified reliability penalties that compress the effective policy set. Throughout, we have argued that systemic niche construction — modifying food environments so that sustainable options become reliably executable — is the primary route to closing the gap.

While individuals can engage in local forms of niche construction (meal preparation, batch cooking, reorganizing the kitchen), they have limited scope to alter the wider reliability of food environments. Systemic niche construction is therefore a key function of policymakers. In this view, bodies such as Defra, the FSA, and OHID are not merely regulators monitoring compliance; they are niche constructors responsible for re-engineering the predictability of the food system (Constant et al., 2022). Their role is not simply to ensure that sustainable food is available, but to stabilize the affordance field by reducing the volatility, friction, and failure costs that make sustainable choices risky (Rietveld and Kiverstein, 2014; Ramstead et al., 2016).

In systems thinking, leverage points are places where relatively small changes in structures, rules, incentives, or feedback loops can produce disproportionate shifts in system behavior (Abson et al., 2017; Meadows, 1999). Within the dietary affordance framework, leverage points are not simply “barrier removal” points. They are points in the food system where intervention changes the precision structure of the affordance field and creates the conditions for sustainable options to build competing policy priors through supported repetition. This means increasing pragmatic value *V*_*i,c*_ (making sustainable outcomes more aligned with the agent’s preferences), increasing predictive precision *P*_*i,c*_ (making sustainable actions more reliably executable), and building policy priors *H*_*i,c*_ for sustainable options (through positive early experiences and repeated successful enactment), while where appropriate weakening the policy priors of unsustainable defaults. A sustainable option becomes behaviorally powerful when it is not only desirable or morally endorsed, but also reliably executable and increasingly habitual under real-world constraints. The design question therefore shifts from “How do we persuade people to choose differently?” to “How do we redesign food environments so that the sustainable policy is the safest, easiest, most predictable, and most habitual option available?”

### The Dietary Precision Matrix

**FIGURE 3:**
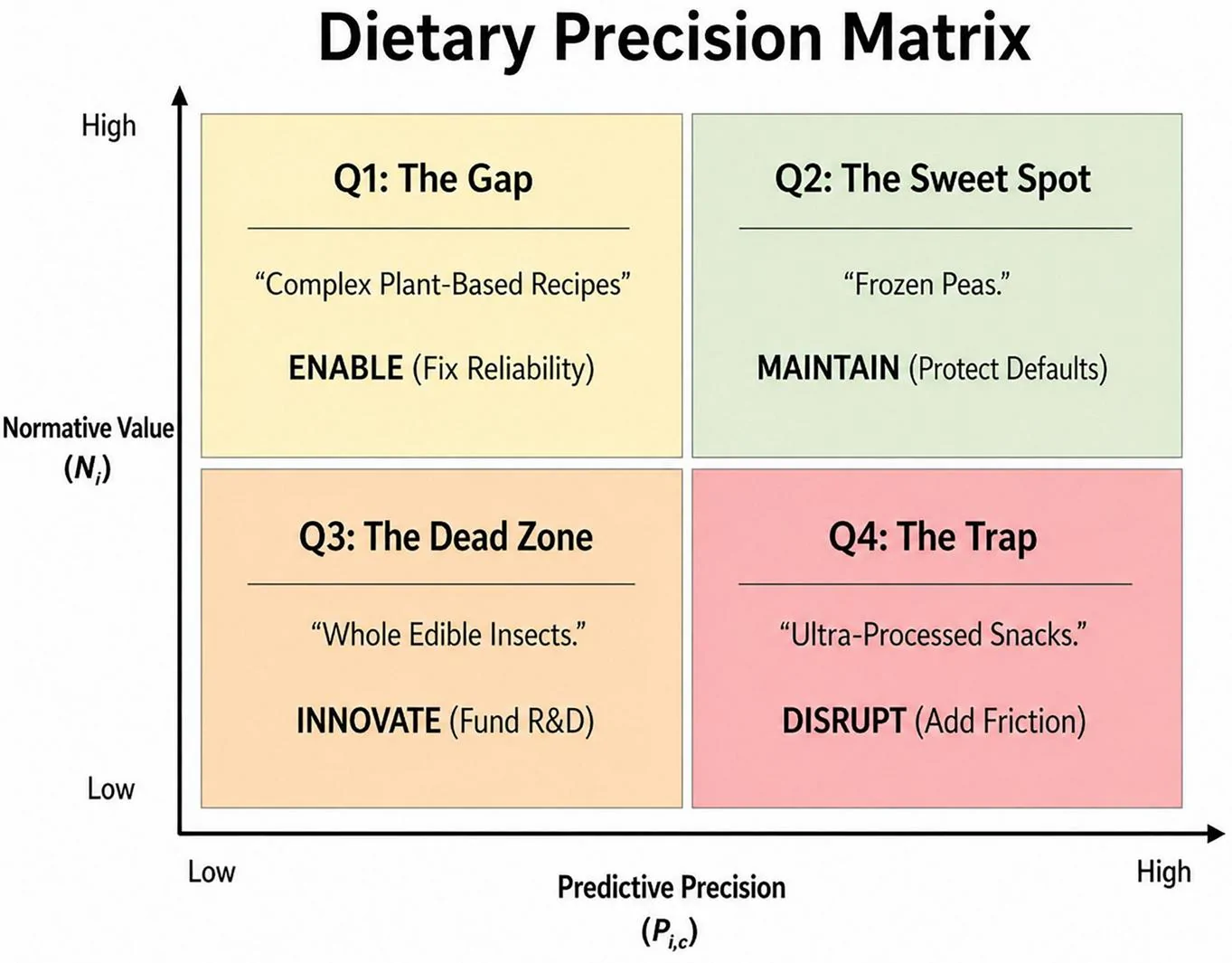
The Dietary Precision Matrix: a framework for policy intervention. The matrix maps food options along two axes: normative value on the Y-axis, reflecting alignment with health and sustainability goals, and predictive precision on the X-axis, reflecting reliability and ease of enactment. Normative value *N*_*i*_ is a policy construct representing the institutional perspective on what agents should prefer; predictive precision *P*_*i,c*_ sits within the active inference framework as the inverse of combined execution uncertainty. The matrix is therefore a hybrid diagnostic tool, combining a societal evaluation of desirability with a behavioral evaluation of feasibility. Q1: The Gap (High Normative Value, Low Precision) captures aspirational foods, such as complex recipes, that fail because they carry high execution uncertainty. Policy: ENABLE by fixing reliability. Q2: The Sweet Spot (High Normative Value, High Precision) captures sustainable habits, such as frozen peas. Policy: MAINTAIN by protecting these defaults. Q3: The Dead Zone (Low Normative Value, Low Precision) captures failed or unattractive products. Policy: INNOVATE through reformulation or research and development. Q4: The Trap (Low Normative Value, High Precision) captures unsustainable habitual defaults, such as ultra-processed foods. Policy: DISRUPT by adding friction or reducing structural support while building high-precision alternatives in Q2. Note that the matrix plots Normative Value *N*_*i*_ against Predictive Precision *P*_*i,c*_, whereas observed behavior is governed by the relative affordance weight: Ã_*i*,*c*_ = *H*_*i*,*c*_⋅exp(γ_*c*_⋅*S*_*i*,*c*_) which additionally incorporates the policy prior *H*_*i,c*_. Because the policy prior strengthens through repeated selection independently of reported enjoyment — consistent with the dissociation between “wanting” and “liking” (Berridge and Robinson, 1998, 2016) — behavior can persist in Q4 even when satisfaction is modest. Sustainable behavior is most reliably sustained in Q2, where both normative value and predictive precision are high and where supported repetition can build sustainable policy priors.

To apply this logic in practice, we introduce the Dietary Precision Matrix (Figure 3) as a diagnostic tool for identifying policy mismatches. The matrix maps food options or behaviors along two dimensions. The first is normative value *N_i_*: the extent to which an option aligns with public goals such as health, sustainability, nutritional adequacy, or reduced environmental impact. The second is predictive precision *P*_*i,c*_: the extent to which that option can be reliably enacted in a specific context, given constraints on price, availability, preparation time, skills, equipment, household acceptance, and fallback options. It is important to note that these two dimensions have different theoretical origins. Normative value is a policy construct introduced from outside the active inference formalism - it represents the institutional perspective on what agents should prefer. Predictive precision sits within the active inference framework as the inverse of combined execution uncertainty. The matrix is therefore a hybrid diagnostic tool, combining a societal evaluation of desirability with a behavioral evaluation of feasibility.

The key point is that normative value and predictive precision are not the same. Policymakers may judge an option to be high value because it is healthy or sustainable, but the consumer may still experience it as low precision if it is expensive, hard to prepare, inconsistently available, socially risky, or likely to fail. Conversely, many unhealthy or unsustainable options may have low normative value but high predictive precision: they are cheap, familiar, shelf-stable, easy to prepare, and reliably accepted (Hall et al., 2019; Leung et al., 2022). The matrix therefore distinguishes between what a food system ought to promote and what it currently makes easy to do.

Q1: The Gap contains options with high normative value but low predictive precision. These are the aspirational foods or behaviors that policy often wants people to adopt, such as cooking complex plant-based meals from scratch, but which may be too costly, time-consuming, volatile, or failure-prone in practice. The intervention imperative is to ENABLE. The task is not primarily to increase motivation, but to increase reliability: reduce preparation burden, stabilize cost, improve availability, lower household rejection risk, and provide fallback support. In this quadrant, information campaigns are unlikely to be sufficient because the problem is not that people fail to value the outcome; it is that the action does not reliably work in context.

Q2: The Sweet Spot contains options with high normative value and high predictive precision. These are the sustainable defaults: options that are both desirable from a public health or sustainability perspective and easy to enact. Examples might include affordable frozen vegetables, tinned pulses, reliable school or workplace meals, or menu options that are sustainable but familiar, filling, and consistently available. The intervention imperative is to MAINTAIN. These options should be protected from price volatility, stock instability, quality variation, or policy changes that would reduce their reliability.

Q3: The Dead Zone contains options with low normative value and low predictive precision. These are products or behaviors that are neither strongly aligned with public goals nor reliably attractive or executable for consumers. The intervention imperative is to INNOVATE. Rather than prematurely subsidizing or scaling these options, policy and industry should improve their basic value proposition and reliability before expecting them to compete. Whole edible insects, for example, may currently sit in this quadrant for many consumers: even if they have sustainability potential, they may lack familiarity, acceptability, preparation knowledge, and reliable culinary integration.

Q4: The Trap contains options with low normative value but high predictive precision. These are the unsustainable habits that dominate because they work. Ultra-processed snacks, fast food, and familiar ready meals often sit here: they are predictable, highly available, low effort, and reliably rewarding, even when they conflict with health or sustainability goals (Hall et al., 2019; Leung et al., 2022). This also aligns with work showing that “wanting” and “liking” can dissociate (Berridge and Robinson, 1998, 2016; Dai et al., 2010). In the present framework, this dissociation is captured by the policy prior *H*_*i,c*_: repeated selection strengthens the prior independently of reported enjoyment, so that cue-triggered habitual selection can persist even when the agent does not rate the option highly on satisfaction. The intervention imperative is to DISRUPT, but carefully. The aim is not simply to remove these options, which may harm constrained households if no viable alternative exists, but to reduce their dominance while building high-precision alternatives in Q2. Otherwise, policy risks shifting consumers from one high precision but undesirable option to another.

The matrix highlights two primary pathways for intervention. The first is horizontal stabilization: moving options from Q1 to Q2 by increasing predictive precision while preserving normative value. This might involve making sustainable foods cheaper, easier to prepare, more consistently stocked, more acceptable to households, or supported by reliable fallback options. The second is vertical improvement: moving options from Q4 to Q2 by improving normative value without undermining precision. This is the logic of “stealth” reformulation, menu optimization, or product reformulation, where the aim is to improve the health or sustainability profile of existing high-precision habits while preserving familiarity, convenience, satiety, and reliability.

This framing also helps policymakers avoid two common errors. The first is the Diagonal Fallacy: asking consumers to jump directly from high-precision unsustainable habits in Q4 to low-precision sustainable aspirations in Q1. This asks people to trade certainty for uncertainty, often under conditions where uncertainty is precisely what they cannot afford. The second is Precision Substitution: restricting or taxing one high-precision unsustainable option without providing a viable high-precision sustainable alternative. In that case, consumers may shift sideways to another reliable but undesirable option rather than upward into Q2. For example, a tax on meat that does not improve the affordability, convenience, and reliability of sustainable substitutes may shift some consumers toward cheaper ultra-processed foods rather than fresh, sustainable meals (Klenert et al., 2023).

### Applying the matrix to policy domains

The Dietary Precision Matrix can be used to reinterpret familiar policy domains. We focus on three examples: fiscal policy, urban planning, and digital choice architecture. These are illustrative rather than exhaustive, but they show how the framework moves from general systems language to specific intervention targets.

#### Fiscal policy: from changing cost to stabilizing reliability

Fiscal policy typically aims to alter behavior by changing relative prices. Taxes on carbon-intensive foods, red meat, or sugar-sweetened products are designed to make undesirable options less attractive, while subsidies are designed to make healthier or more sustainable options more affordable (Springmann et al., 2018; Klenert et al., 2023). This approach is important, but an affordance perspective suggests that mean price is only part of the decision problem. For constrained households, volatility may matter as much as, or more than, average cost. A food that is affordable on average may still be low precision if its price varies week to week, if discounts are short-lived, or if the household cannot predict whether it will remain within budget across a pay cycle. Food price volatility is also a recognized driver of welfare risk (Gilbert and Morgan, 2010).

The leverage point is therefore not only cost, but price stability and reliability. A carbon tax on meat, for example, may reduce the attractiveness of a high-impact food, but if the available alternatives remain high variance, the policy may not produce the intended shift toward fresh sustainable ingredients. Fresh vegetables, legumes, or plant-based meals may still carry spoilage risk, preparation burden, uncertain satiety, or household rejection (Jabs and Devine, 2006; Venn and Strazdins, 2017). Under constraint, consumers may instead substitute toward another high-precision option: cheaper ready meals, shelf-stable snacks, or ultra-processed products. This is the risk of precision substitution.

An affordance-based fiscal policy would therefore subsidize reliability, not only health or sustainability in the abstract. This may mean supporting frozen vegetables, tinned pulses, minimally processed plant-based meals, and other formats that combine sustainability with shelf stability, predictable preparation time, low waste risk, and household acceptability. Constrained households tend to rely more on shelf-stable, ready-to-eat formats, including ultra-processed foods, because they minimize day-to-day risk and effort (Leung et al., 2022). The aim is therefore to move sustainable options from Q1 to Q2 by making them reliably affordable and executable. In practical terms, the question becomes not simply “Are sustainable foods cheaper?” but “Are sustainable foods reliably affordable when households need them, and do they remain viable when plans fail?”

#### Urban planning: from physical access to predictive reliability

Urban planning has often focused on physical proximity: whether people live near supermarkets, healthy food outlets, or fresh produce retailers. This has generated important work on food deserts and food swamps (Cooksey-Stowers et al., 2017). However, the affordance framework suggests that access is not equivalent to reliability. A healthy or sustainable food outlet may be nearby but still fail to function as a high-precision policy if opening hours are inconsistent, stock is unreliable, prices vary, quality is unpredictable, or fallback options are poor.

The leverage point is therefore predictive reliability across daily activity spaces. Fast-food chains and convenience stores often dominate not only because they are nearby, but because they are operationally precise. They offer consistent hours, recognizable products, predictable prices, visible signage, and low search costs. By contrast, sustainable food environments such as farmers’ markets, independent greengrocers, community food schemes, or some canteen settings may be experienced as higher variance: seasonal stock, limited hours, variable quality, and uncertain availability (Mack and Tong, 2015). For an agent minimizing expected risk under time pressure, this uncertainty can make the sustainable option less viable even when it is geographically accessible.

This logic is illustrated by Flynn et al. (2025), who optimized a university canteen menu to reduce its carbon footprint by 30%. The intervention preserved the most preferred dish as a reliable fallback while pairing lower-carbon options with familiar, acceptable menu structures. From the perspective of the Dietary Precision Matrix, this is a Q4-to-Q2 vertical shift: the normative value of the food environment improved while predictive precision was maintained. Because the intervention did not require students to trade familiarity, satiety, or fallback security for sustainability, the sustainable policy could be enacted with minimal hedonic or metabolic surprise.

Urban planning interventions should therefore ask whether sustainable food provision is dependable enough to compete with high-precision incumbents. This includes reliable opening times, stable supply, visible and familiar sustainable options, safe and convenient routes, and fallback provision when the preferred option is unavailable. Planning may also reduce the precision of unhealthy defaults, for example by limiting the density, visibility, or cue intensity of fast-food outlets around schools, workplaces, or commuting routes (Burgoine et al., 2014; Brown et al., 2022; Yau et al., 2022). However, disruption should be paired with Q2 alternatives. Reducing exposure to Q4 options without increasing the reliability of Q2 options risks leaving constrained households with fewer viable choices.

The planning question therefore shifts from “Is healthy food nearby?” to “Can people depend on sustainable food options across the real geography of their lives?” This includes home, work, school, commuting routes, shift schedules, and caregiving routines. In this sense, urban planning becomes a form of niche construction: it shapes not only what is available in space, but which policies can be enacted reliably over time (Laland et al., 2016; Constant et al., 2022).

#### Digital choice architecture: from information to low-friction defaults

Digital retail and food-ordering environments increasingly shape dietary behavior. Online grocery platforms, delivery apps, and institutional ordering systems do not simply present options; they structure the policy set. Search rankings, default baskets, repeat-order functions, suggested swaps, promotions, and delivery substitutions all influence which options are easiest to select under time pressure. Previous work shows that online grocery environments can shape habitual patterns and reduce or increase reflective engagement depending on interface design (Huyghe et al., 2017).

Traditional digital interventions often focus on information: labels, prompts, sustainability scores, or warnings. These may increase knowledge or value, but they do not necessarily increase precision. A consumer may learn that a lower-carbon meal is preferable, but still default to a familiar basket if finding, comparing, and assembling the sustainable alternative requires additional time and uncertainty. In active inference terms, the existing repeat order is a high-precision policy: it is known, fast, and unlikely to fail. This overlaps with, but reframes, behavioral economic accounts of defaults and choice architecture, which often emphasize status quo bias or inertia (Thaler et al., 2013).

The leverage point is therefore default reliability and interaction cost. Digital systems can move sustainable options toward Q2 by reducing search burden, preserving familiarity, and making sustainable substitutions low risk. For example, platforms could offer default sustainable swaps that match price, satiety, preparation time, and household preferences; provide reliable meal bundles rather than isolated ingredients; or flag substitutions that preserve the intended meal rather than simply replacing an out-of-stock item with the nearest product. These interventions work not by tricking consumers, but by offloading cognitive and logistical costs onto the environment.

However, digital defaults must be reliable after selection. A sustainable default that is unexpectedly expensive, poor in satiety, frequently out of stock, or rejected by the household will rapidly lose precision. The design standard is therefore not simply whether a digital prompt increases immediate uptake, but whether the selected option remains a low-surprise experience after purchase and consumption (Friston et al., 2016; Parr and Friston, 2019). In matrix terms, digital choice architecture should not merely push consumers toward Q1 aspirations. It should help build Q2 defaults: sustainable options that are easy to select, reliably delivered, and successful enough to be repeated.

### Summary

Across fiscal policy, urban planning, and digital choice architecture, the same principle applies. Effective leverage points are not simply those that increase motivation or make sustainable options nominally available. They are interventions that alter the precision structure of the affordance field and create the conditions for sustainable options to build competing policy priors through supported repetition. Fiscal policy can stabilize reliable sustainable substitutes. Urban planning can make sustainable provision dependable across daily routines. Digital architecture can reduce search and planning costs while preserving post-choice reliability. Together, these interventions aim to close the intention–behavior gap by making sustainable policies not only preferred, but feasible, low-risk, and repeatable.

## Operationalizing the framework: from theory to testable predictions

Having identified where interventions should target the affordance field, we now turn to how these leverage points can be measured and tested empirically. The dietary affordance framework is intended as a reduced-form computational framework, but it generates specific empirical predictions that can be tested without estimating a full generative model. Its key methodological shift is from measuring “access” as a static property of environments to measuring “precision” as the reliability with which an action can be enacted in context. This means asking not only whether sustainable foods are available, affordable, or valued, but whether they are stable, predictable, low-risk, and repeatable under real-world constraints.

Predictive precision *P*_*i,c*_ can be operationalized using observable environmental and behavioral variables. These include price volatility, stock-out frequency, preparation-time variance, spoilage risk, household rejection risk, fallback availability, and the cost of failed enactment. Such indicators can be measured through transaction panels, loyalty-card data, repeated store audits, online stock records, ecological momentary assessment, time-use diaries, and natural experiments such as price shocks, supply disruptions, or policy changes. Policy priors *H*_*i,c*_ can be approximated through repeat-purchase frequency or habitual purchase measures from transaction data. Pragmatic value *V*_*i,c*_ can be estimated through stated preference measures, willingness-to-pay tasks, or goal endorsement scales, allowing researchers to test the independent and interactive contributions of value, precision, and habit to observed dietary behavior.

Importantly, testing the framework does not require immediate estimation of a full Bayesian generative model. The reduced-form affordance equation:


$${\tilde{A}}_{i,c}=H_{i,c}\cdot \text{exp}\,\left(\gamma_{c}\cdot S_{i,c}\right)$$

where


$$S_{i,c}=V_{i,c}+P_{i,c}$$

generates empirical predictions that can be tested using interaction models, threshold models, mediation analysis, quasi-experimental designs, and longitudinal data.

Together, these hypotheses distinguish the dietary affordance framework from approaches that focus primarily on motivation, information, or physical access. The central empirical claim is that sustainable behavior should emerge when valued options become reliable enough to remain within the feasible policy set. Empirical work can therefore validate, refine, or falsify the framework by testing whether changes in predictive precision explain when sustainable intentions become sustainable habits.

This also clarifies the contribution of the reduced-form affordance equation. The equation is not intended to replace a fully specified computational model. Rather, it provides an empirical scaffold for identifying when and where sustainable options fail. If the hypotheses above are supported, they would show that food environments shape behavior not only by changing what people want, know, or can physically access, but by altering the reliability of the policies available to them. If they are not supported, the framework can be refined by identifying which dimensions of precision matter most, for whom, and under what constraints.

## Future directions: from reduced-form heuristic to computational model

The present framework uses a reduced-form approximation of active inference policy selection to make the affordance account empirically tractable. While we have shown (Supplementary Material 2) that this approximation preserves the algebraic structure of the standard policy selection mechanism (multiplicative policy prior, additive policy score inside the exponent, softmax normalization), it does so by collapsing several architecturally distinct components of a full generative model into summary scalars. Future work can extend the framework by unpacking these components and testing whether finer-grained decompositions improve explanatory or predictive power.

First, predictive precision *P*_*i,c*_, as defined here, combines sources of uncertainty that would be formally separated in a fully specified active inference model. Outcome ambiguity (the entropy of the likelihood mapping from hidden states to observations), transition uncertainty (the reliability with which actions produce intended state changes, encoded in the *B* matrices of a discrete state-space model), and external volatility (price fluctuation, stock instability, spoilage risk) each contribute differently to expected free energy and may respond differently to intervention. For example, a subsidy reduces price volatility but does not address transition uncertainty, such as whether the agent can reliably execute the cooking steps, while a meal kit reduces transition uncertainty but may leave price volatility intact. Decomposing *P*_*i,c*_ into these constituent sources would allow researchers to test which dimensions of precision matter most, for whom, and under what constraints, and would enable more targeted policy recommendations. A natural next step would be to construct a discrete state-space generative model of a simplified dietary choice scenario and compare its predictions with those of the reduced form, identifying the conditions under which the aggregated *P*_*i,c*_ diverges from a decomposed treatment.

Second, pragmatic value *V*_*i,c*_ is treated here as a scalar summary of goal alignment, whereas in the full active inference formalism, the corresponding risk term is a Kullback–Leibler divergence between the predicted outcome distribution under a policy and the agent’s prior preference distribution. This distributional structure carries information that a scalar cannot: two options with identical mean alignment but different variance profiles would receive the same *V*_*i,c*_ score in the present framework but different risk evaluations in a full model. Empirically, this distinction may matter in contexts where agents face asymmetric downside risk - for example, when a sustainable meal that is usually satisfying occasionally fails badly versus one that is consistently mediocre. Future empirical work could test whether distributional features of outcome alignment predict choice above and beyond mean value, which would indicate whether the scalar approximation is sufficient or whether a richer representation is needed.

Third, the context-dependence of both the policy prior *H*_*i,c*_ and policy-selection precision γ_*c*_ implies a hierarchical generative model in which context is inferred and used to retrieve context-appropriate priors and precision settings. In the simplest active inference formulations, the policy prior *E*(π) is a fixed vector and γ is a global parameter estimated through a separate precision-inference step (Friston et al., 2016). Context-dependent versions are available in the hierarchical active inference literature (Pezzulo et al., 2018), but they require specifying how contexts are represented, how context inference proceeds, and how policy priors are structured across contexts. The present framework leaves this architecture implicit. Specifying it would allow the model to address important empirical phenomena, such as how a person who cooks sustainably at home may default to processed food at work, and how context boundaries are learned and maintained.

Fourth, although we reintroduce epistemic value when discussing the initiation problem, the present framework does not model the dynamics of belief updating - how outcome beliefs and policy priors evolve over repeated encounters with a food option. Active inference provides a natural apparatus for this through Bayesian belief updating and Dirichlet concentration parameters that accumulate evidence (Da Costa et al., 2020). Formalizing the learning dynamics would allow the framework to make quantitative predictions about the rate of habit formation, the number of successful encounters needed to establish a competing policy prior, and the conditions under which a single negative experience can durably suppress future selection. These predictions are central to the “race against precision” argument and would benefit from explicit computational treatment. Finally, the present framework models individual policy selection and does not formally address multi-agent dynamics, social learning, or the co-evolution of consumer behavior and market supply. In practice, the affordance field is shaped by aggregate consumer behavior - retailers stock what sells, and norms emerge from shared practice. Extending the model to incorporate these feedback dynamics, potentially through multi-agent active inference or evolutionary game-theoretic frameworks, would allow the framework to address how sustainable norms can reach tipping points and how market feedback loops can lock in or disrupt dietary habits at population scale. These extensions would move the framework from a structured heuristic toward a testable computational model, while preserving the core insight that behavior reflects policy selection under constraint rather than motivational deficit.

## Conclusion: designing for the path of least resistance

This paper began with a persistent disconnect: why do intentions explain substantially more variance in what people say they plan to do than in what they actually do? In sustainable eating, this gap is especially consequential because many people endorse health and sustainability goals while continuing to rely on convenient, energy-dense, and often unsustainable foods. We have argued that this should not be understood primarily as a failure of self-regulation. From an active inference perspective, the gap can instead be understood as a predictable outcome of policy selection under constraint. In volatile food environments, the system selects policies that minimize expected risk, uncertainty, and costly surprise. Ultra-processed foods, ready meals, and takeaway options often function as high-reliability policies with strong policy priors: they are predictable in cost, preparation time, sensory outcome, and satiety, and they have been reinforced through years of successful repetition. By contrast, sustainable intentions may remain weakly supported higher-level goals when the actions required to realize them carry risks of spoilage, price volatility, time overrun, or household rejection, and when no policy prior has been built through repeated successful enactment.

The central contribution of the paper is the concept of dietary affordances: the set of food-related actions that are realistically available and achievable for a person in context. We formalize this through the relative affordance weight:


$${\tilde{A}}_{i,c}=H_{i,c}\cdot \text{exp}\,\left(\gamma_{c}\cdot S_{i,c}\right)$$

where the policy score


$$S_{i,c}=V_{i,c}+P_{i,c}$$

combines pragmatic value and predictive precision, and selection probability is determined by normalization across the feasible affordance field. This shifts the policy question from “Is the sustainable option available?” to “Is the sustainable action reliably executable for this person, in this context, and has it been enacted often enough to become habitual?” The framework highlights three intervention-relevant insights. First, information campaigns may increase value, concern, or goal salience, but often fail when they leave enactment precision unchanged and do not address the policy priors of incumbent habits. Second, timing matters because early encounters shape both predictive precision and policy priors: unreliable first experiences can prevent the formation of sustainable habits. Third, initiation and continuation are distinct problems: initial uptake requires safe exploration; sustained change requires repeated reliable enactment that builds policy priors. The framework therefore generates testable predictions, including threshold effects, Intention × Precision interactions, precision substitution, and the hypothesis that operational reliability may predict behavior better than physical proximity alone.

This reframing addresses a specific gap in the current policy landscape. Despite widespread recognition that information and labeling campaigns are insufficient, the alternative - a “systems approach” - has largely remained a descriptive aspiration rather than a framework that specifies what to change and why it should work. The dietary affordance framework provides this missing middle layer: a mechanistic account that connects environmental conditions to behavioral outcomes with enough specificity to generate testable predictions and actionable intervention targets. Crucially, by demonstrating that unsustainable dietary behavior is computationally rational given the environments in which it occurs, the framework shifts the locus of responsibility from individual consumers to the institutions that design and govern food systems - the level at which structural change is both possible and necessary.

The policy implication is direct: we cannot inform our way out of a structural crisis. Closing the sustainable eating intention–behavior gap requires engineering reliability and building sustainable habits. Fiscal policy should not only change average prices but stabilize affordability and subsidize reliable sustainable formats. Urban planning should move beyond proximity and design for operational consistency across daily routines. Digital choice architecture should reduce search and planning costs while preserving post-choice reliability. Across these domains, the goal is structural alignment: making sustainable choices the safest, easiest, and most predictable policies available, and ensuring that they are enacted often enough to build competing policy priors. A food system that relies on individual motives and self-regulation to mitigate a climate crisis is a system designed to fail. By aligning environmental affordances with the brain’s drive to minimize uncertainty, sustainable habits can emerge not through exhausting self-regulation, but as the natural outcome of a well-designed system.

## Data Availability

The original contributions presented in this study are included in the article/Supplementary material, further inquiries can be directed to the corresponding author.

## References

[B1] Abson D. J. Fischer J. Leventon J. Newig J. Schomerus T. Vilsmaier U. et al. (2017). Leverage points for sustainability transformation. *Ambio* 46 30–39. 10.1007/s13280-016-0800-y 27344324 PMC5226895

[B2] Ajzen I. (1991). The theory of planned behavior. *Organ. Behav. Hum. Decis. Process.* 50 179–211. 10.1016/0749-5978(91)90020-T

[B3] Armitage C. J. Conner M. (2001). Efficacy of the theory of planned behaviour: A meta-analytic review. *Br. J. Soc. Psychol.* 40 471–499. 10.1348/014466601164939 11795063

[B4] Benton D. Young H. A. (2024). There is more to nutrition and cardiovascular disease than ultra-processing. *Health* 48:101141. 10.1016/j.lanepe.2024.101141 PMC11980616 40206219

[B5] Berridge K. C. Robinson T. E. (1998). What is the role of dopamine in reward: Hedonic impact, reward learning, or incentive salience? *Brain Res. Rev.* 28 309–369. 10.1016/S0165-0173(98)00019-8 9858756

[B6] Berridge K. C. Robinson T. E. (2016). Liking, wanting, and the incentive-sensitization theory of addiction. *Am. Psychol.* 71 670–679. 10.1037/amp0000059 27977239 PMC5171207

[B7] Blanke J. Billieux J. Vögele C. (2022). Healthy and sustainable food shopping: A survey of intentions and motivations. *Front. Nutr.* 9:855577. 10.3389/fnut.2022.742614 PMC8924458 35308289

[B8] Brown H. Xiang H. Albani V. Goffe L. Akhter N. Lake A. et al. (2022). No new fast-food outlets allowed! Evaluating the effect of planning policy on the local food environment in the North East of England. *Soc. Sci. Med.* 306:115126. 10.1016/j.socscimed.2022.115126 35724588

[B9] Burgoine T. Forouhi N. G. Griffin S. J. Wareham N. J. Monsivais P. (2014). Associations between exposure to takeaway food outlets, takeaway food consumption, and body weight in Cambridgeshire, UK: Population based, cross sectional study. *BMJ* 348:g1464. 10.1136/bmj.g1464 24625460 PMC3953373

[B10] Coates J. Frongillo E. Rogers B. Webb P. Wilde P. Houser R. (2006). Commonalities in the experience of household food insecurity across cultures: What are measures missing? *J. Nutr.* 136 1438S–1448S. 10.1093/jn/136.5.1438S 16614441

[B11] Conner M. Norman P. (2022). Understanding the intention-behavior gap: The role of intention strength. *Front. Psychol.* 13:923464. 10.3389/fpsyg.2022.923464 35992469 PMC9386038

[B12] Constant A. Clark A. Kirchhoff M. Friston K. J. (2022). Extended active inference: Constructing predictive cognition beyond skulls. *Mind Lang.* 37 373–394. 10.1111/mila.12330 35875359 PMC9292365

[B13] Cooksey-Stowers K. Schwartz M. B. Brownell K. D. (2017). Food swamps predict obesity rates better than food deserts in the United States. *Int. J. Environ. Res. Public Health* 14:1366. 10.3390/ijerph14111366 29135909 PMC5708005

[B14] Crippa M. Solazzo E. Guizzardi D. Monforti-Ferrario F. Tubiello F. N. Leip A. (2021). Food systems are responsible for a third of global anthropogenic GHG emissions. *Nat. Food* 2 198–209. 10.1038/s43016-021-00225-9 37117443

[B15] Culliford A. Bradbury J. (2020). A cross-sectional survey of the readiness of consumers to adopt an environmentally sustainable diet. *Nutr. J.* 19:138. 10.1186/s12937-020-00644-7 33298065 PMC7727219

[B16] Da Costa L. Parr T. Sajid N. Veselic S. Neacsu V. Friston K. (2020). Active inference on discrete state-spaces: A synthesis. *J. Math. Psychol.* 99:102447. 10.1016/j.jmp.2020.102447 33343039 PMC7732703

[B17] Dai X. Brendl C. M. Ariely D. (2010). Wanting, liking, and preference construction. *Emotion* 10 324–334. 10.1037/a0017549 20515222

[B18] Day R. L. Laland K. N. Odling-Smee F. J. (2003). Rethinking adaptation: The niche-construction perspective. *Perspect. Biol. Med.* 46 80–95. 10.1353/pbm.2003.0003 12582272

[B19] De Schutter O. Jacobs N. Clément C. (2020). A ‘Common Food Policy’ for Europe: How governance reforms can spark a shift to healthy diets and sustainable food systems. *Food Policy* 96:101849. 10.1016/j.foodpol.2020.101849

[B20] ElHaffar G. Durif F. Dubé L. (2020). Towards closing the attitude-intention-behavior gap in green consumption: A narrative review of the literature and an overview of future research directions. *J. Clean. Prod.* 275:122556. 10.1016/j.jclepro.2020.122556

[B21] Evans J. S. B. T. Stanovich K. E. (2013). Dual-process theories of higher cognition: Advancing the debate. *Perspect. Psychol. Sci.* 8 223–241. 10.1177/1745691612460685 26172965

[B22] Feldman H. Friston K. J. (2010). Attention, uncertainty, and free-energy. *Front. Hum. Neurosci.* 4:215. 10.3389/fnhum.2010.00215 21160551 PMC3001758

[B23] FitzGerald T. H. B. Dolan R. J. Friston K. (2015). Dopamine, reward learning, and active inference. *Front. Comput. Neurosci.* 9:136. 10.3389/fncom.2015.00136 26581305 PMC4631836

[B24] Flynn A. N. Takahashi T. Sim A. Brunstrom J. M. (2025). Dish swap across a weekly menu can deliver health and sustainability gains. *Nat. Food* 6 843–847. 10.1038/s43016-025-01218-8 40789949 PMC12454130

[B25] Frank P. Brock C. (2018). Bridging the intention–behavior gap among organic grocery customers: The crucial role of point-of-sale information. *Psychol. Mark.* 35 586–602. 10.1002/mar.21108

[B26] Friston K. (2010). The free-energy principle: A unified brain theory? *Nat. Rev. Neurosci.* 11 127–138. 10.1038/nrn2787 20068583

[B27] Friston K. J. Shiner T. FitzGerald T. Galea J. M. Adams R. Brown H. et al. (2012). Dopamine, affordance and active inference. *PLoS Comput. Biol.* 8:e1002327. 10.1371/journal.pcbi.1002327 22241972 PMC3252266

[B28] Friston K. FitzGerald T. Rigoli F. Schwartenbeck P. Pezzulo G. (2017). Active inference: A process theory. *Neural Comput.* 29 1–49. 10.1162/NECO_a_00912 27870614

[B29] Friston K. FitzGerald T. Rigoli F. Schwartenbeck P. O’Doherty J. Pezzulo G. (2016). Active inference and learning. *Neurosci. Biobehav. Rev.* 68 862–879. 10.1016/j.neubiorev.2016.06.022 27375276 PMC5167251

[B30] Friston K. Rigoli F. Ognibene D. Mathys C. Fitzgerald T. Pezzulo G. (2015). Active inference and epistemic value. *Cogn. Neurosci.* 6 187–214. 10.1080/17588928.2015.1020053 25689102

[B31] Friston K. Schwartenbeck P. Fitzgerald T. Moutoussis M. Behrens T. Dolan R. J. (2013). The anatomy of choice: Active inference and agency. *Front. Hum. Neurosci.* 7:598. 10.3389/fnhum.2013.00598 24093015 PMC3782702

[B32] Gibson J. J. (1977). “The theory of affordances,” in *Perceiving, Acting and Knowing: Towards an Ecological Psychology*, eds Shaw R. Bransford J. (Hillsdale, NJ: Erlbaum), 67–82.

[B33] Gilbert C. L. Morgan C. W. (2010). Food price volatility. *Philos. Trans. R. Soc. Lond. B Biol. Sci.* 365 3023–3034. 10.1098/rstb.2010.0139 20713400 PMC2935118

[B34] Groening C. Sarkis J. Zhu Q. (2018). Green marketing consumer-level theory review: A compendium of applied theories and further research directions. *J. Clean. Prod.* 172 1848–1866. 10.1016/j.jclepro.2017.12.002

[B35] Hall K. D. Ayuketah A. Brychta R. Cai H. Cassimatis T. Chen K. Y. et al. (2019). Ultra-processed diets cause excess calorie intake and weight gain: An inpatient randomized controlled trial of ad libitum food intake. *Cell Metab.* 30 67–77.e3. 10.1016/j.cmet.2019.05.008 31105044 PMC7946062

[B36] Hill R. Hughes K. Cresswell K. Ford K. Bellis M. (2023). *The Rising Cost of Living and Health and Wellbeing in Wales: A National Survey.* Wrexham: Public Health Wales.

[B37] Huyghe E. Verstraeten J. Geuens M. Van Kerckhove A. (2017). Clicks as a healthy alternative to bricks: How online grocery shopping reduces vice purchases. *J. Mark. Res.* 54 61–74. 10.1509/jmr.14.0490

[B38] Jabs J. Devine C. M. (2006). Time scarcity and food choices: An overview. *Appetite* 47 196–204. 10.1016/j.appet.2006.02.014 16698116

[B39] Joshi Y. Rahman Z. (2015). Factors affecting green purchase behaviour and future research directions. *Int. Strateg. Manag. Rev.* 3 128–143. 10.1016/j.ism.2015.04.001

[B40] Kahneman D. (2011). *Thinking, Fast and Slow.* New York, NY: Farrar, Straus and Giroux.

[B41] Kelly B. Jewell J. (2018). *What Is the Evidence on the Policy Specifications, Development Processes and Effectiveness of Existing Front-of-Pack Food Labelling Policies in the WHO European Region?.* Copenhagen: WHO Regional Office for Europe. 30484994

[B42] Klenert D. Funke F. Cai M. (2023). Meat taxes in Europe can be designed to avoid overburdening low-income consumers. *Nat. Food* 4 894–901. 10.1038/s43016-023-00849-z 37783791 PMC10589082

[B43] Laland K. N. Sterelny K. (2006). Perspective: Seven reasons (not) to neglect niche construction. *Evolution* 60 1751–1762. 10.1111/j.0014-3820.2006.tb00520.x 17089961

[B44] Laland K. Matthews B. Feldman M. W. (2016). An introduction to niche construction theory. *Evol. Ecol.* 30 191–202. 10.1007/s10682-016-9821-z 27429507 PMC4922671

[B45] Leung C. W. Fulay A. P. Parnarouskis L. Martinez-Steele E. Gearhardt A. N. Wolfson J. A. (2022). Food insecurity and ultra-processed food consumption: The modifying role of participation in the Supplemental Nutrition Assistance Program (SNAP). *Am. J. Clin. Nutr.* 116 197–205. 10.1093/ajcn/nqac049 35199832 PMC9257471

[B46] Lobo L. Heras-Escribano M. Travieso D. (2018). The history and philosophy of ecological psychology. *Front. Psychol.* 9:2228. 10.3389/fpsyg.2018.02228 30555368 PMC6280920

[B47] Loopstra R. Reeves A. Taylor-Robinson D. Barr B. McKee M. Stuckler D. (2015). Austerity, sanctions, and the rise of food banks in the UK. *BMJ* 350:h1775. 10.1136/bmj.h1775 25854525

[B48] Mack J. Tong D. (2015). Characterizing the spatial and temporal patterns of farmers’ market visits. *Appl. Geogr.* 63 43–54. 10.1016/j.apgeog.2015.06.005

[B49] Mani A. Mullainathan S. Shafir E. Zhao J. (2013). Poverty impedes cognitive function. *Science* 341 976–980. 10.1126/science.1238041 23990553

[B50] Meadows D. H. (1999). *Leverage Points: Places to Intervene in a System.* Hartland, VT: The Sustainability Institute.

[B51] Meadows J. Montano M. Alfar A. J. K. Başkan ÖY. De Brún C. Hill J. et al. (2024). The impact of the cost-of-living crisis on population health in the UK: Rapid evidence review. *BMC Public Health* 24:561. 10.1186/s12889-024-17940-0 38388342 PMC10882727

[B52] Michie S. Van Stralen M. M. West R. (2011). The behaviour change wheel: A new method for characterising and designing behaviour change interventions. *Implement. Sci.* 6:42. 10.1186/1748-5908-6-42 21513547 PMC3096582

[B53] O’Keefe L. McLachlan C. Gough C. Mander S. Bows-Larkin A. (2016). Consumer responses to a future UK food system. *Br. Food J.* 118 412–428. 10.1108/BFJ-01-2015-0047

[B54] Papies E. K. Barsalou L. W. Rusz D. (2020). Understanding desire for food and drink: A grounded-cognition approach. *Curr. Dir. Psychol. Sci.* 29 193–198. 10.1177/0963721420904958

[B55] Parr T. Friston K. J. (2019). Generalised free energy and active inference. *Biol. Cybern.* 113 495–513. 10.1007/s00422-019-00805-w 31562544 PMC6848054

[B56] Pezzulo G. Rigoli F. Friston K. J. (2018). Hierarchical active inference: A theory of motivated control. *Trends Cogn. Sci.* 22 294–306. 10.1016/j.tics.2018.01.009 29475638 PMC5870049

[B57] Radimer K. L. Olson C. M. Greene J. C. Campbell C. C. Habicht J.-P. (1992). Understanding hunger and developing indicators to assess it in women and children. *J. Nutr. Educ.* 24 36S–44S. 10.1016/S0022-3182(12)80137-3

[B58] Ramstead M. J. D. Veissière S. P. L. Kirmayer L. J. (2016). Cultural affordances: Scaffolding local worlds through shared intentionality and regimes of attention. *Front. Psychol.* 7:1090. 10.3389/fpsyg.2016.01090 27507953 PMC4960915

[B59] Remnant J. Adams J. (2015). The nutritional content and cost of supermarket ready-meals. Cross-sectional analysis. *Appetite* 92 36–42. 10.1016/j.appet.2015.04.069 25963106 PMC4509783

[B60] Rietveld E. Kiverstein J. (2014). A rich landscape of affordances. *Ecol. Psychol.* 26 325–352. 10.1080/10407413.2014.958035

[B61] Rockström J. Thilsted S. H. Willett W. C. Gordon L. J. Herrero M. Hicks C. C. et al. (2025). The EAT–lancet commission on healthy, sustainable, and just food systems. *Lancet* 406 1625–1700. 10.1016/S0140-6736(25)01201-2 41046857

[B62] Romijn A. R. Latulippe M. E. Snetselaar L. Willatts P. Melanson L. Gershon R. et al. (2023). Perspective: Advancing dietary guidance for cognitive health—focus on solutions to harmonize test selection, implementation, and evaluation. *Adv. Nutr.* 14 366–378. 10.1016/j.advnut.2023.02.001 36997091 PMC10201813

[B63] Sallis J. F. Owen N. (2015). “Ecological models of health behavior,” in *Health Behavior: Theory, Research, and Practice*, 5th Edn, eds Glanz K. Rimer B. K. Viswanath K. (San Francisco, CA: Jossey-Bass), 43–64.

[B64] Sheeran P. (2002). Intention—behavior relations: A conceptual and empirical review. *Eur. Rev. Soc. Psychol.* 12 1–36. 10.1080/14792772143000003

[B65] Shove E. Pantzar M. Watson M. (2012). *The Dynamics of Social Practice: Everyday Life and How It Changes.* London: SAGE Publications.

[B66] Springmann M. Clark M. Mason-D’Croz D. Wiebe K. Bodirsky B. L. Lassaletta L. et al. (2018). Options for keeping the food system within environmental limits. *Nature* 562 519–525. 10.1038/s41586-018-0594-0 30305731

[B67] Thaler R. H. Sunstein C. R. Balz J. P. (2013). “Choice architecture,” in *The Behavioral Foundations of Public Policy*, ed. Shafir E. (Princeton, NJ: Princeton University Press), 428–439.

[B68] Trope Y. Liberman N. (2010). Construal-level theory of psychological distance. *Psychol. Rev.* 117 440–463. 10.1037/a0018963 20438233 PMC3152826

[B69] USDA Economic Research Service (2026). *Food Security in the U.S.* Available online at: https://www.ers.usda.gov/topics/food-nutrition-assistance/food-security-in-the-u-s/ (accessed January 15, 2026)

[B70] Vargas C. Brooke D. Hargous C. V. Fernández-Rodríguez R. Bennett R. Jacka F. et al. (2025). Initiatives for advancing planetary health through sustainable food systems: An umbrella review. *Obes. Rev.* 27:e70074. 10.1111/obr.70074 41420417

[B71] Venn D. Strazdins L. (2017). Your money or your time? How both types of scarcity matter to physical activity and healthy eating. *Soc. Sci. Med.* 172 98–106. 10.1016/j.socscimed.2016.10.023 27839899

[B72] Vossel S. Mathys C. Daunizeau J. Bauer M. Driver J. Friston K. J. et al. (2013). Spatial attention, precision, and Bayesian inference: A study of saccadic response speed. *Cereb. Cortex* 24 1436–1450. 10.1093/cercor/bhs418 23322402 PMC4014178

[B73] Willett W. Rockström J. Loken B. Springmann M. Lang T. Vermeulen S. et al. (2019). Food in the anthropocene: The EAT–lancet commission on healthy diets from sustainable food systems. *Lancet* 393 447–492. 10.1016/S0140-6736(18)31788-4 30660336

[B74] Withagen R. De Poel H. J. Araújo D. Pepping G.-J. (2012). Affordances can invite behavior: Reconsidering the relationship between affordances and agency. *New Ideas Psychol.* 30 250–258. 10.1016/j.newideapsych.2011.12.003

[B75] Wood W. Rünger D. (2016). Psychology of habit. *Annu. Rev. Psychol.* 67 289–314. 10.1146/annurev-psych-122414-033417 26361052

[B76] Wrieden W. Halligan J. Goffe L. Barton K. Leinonen I. (2019). Sustainable diets in the UK—developing a systematic framework to assess the environmental impact, cost and nutritional quality of household food purchases. *Sustainability* 11:4974. 10.3390/su11184974

[B77] Yau A. Berger N. Law C. Cornelsen L. Greener R. Adams J. et al. (2022). Changes in household food and drink purchases following restrictions on the advertisement of high fat, salt, and sugar products across the Transport for London network: A controlled interrupted time series analysis. *PLoS Med.* 19:e1003915. 10.1371/journal.pmed.1003915 35176022 PMC8853584

[B78] Young H. A. Gaylor C. M. de-Kerckhove D. Benton D. (2021). Individual differences in sensory and expectation driven interoceptive processes: A novel paradigm with implications for alexithymia, disordered eating and obesity. *Sci. Rep.* 11:10065. 10.1038/s41598-021-89417-8 33980896 PMC8115295

