# Supplementary material for: From intention–behavior gap to dietary affordances: an active inference account of sustainable eating
Source: Front Psychol. 2026 Aug 11;17:1779626. doi: 10.3389/fpsyg.2026.1779626 (PMC13503352; doi:10.3389/fpsyg.2026.1779626)
Supplement: Supplementary file 1 [file Supplementary_file_1.docx]

**Supplementary Material 1: Positioning the Dietary Affordance Framework Relative to Existing Approaches**

Several existing frameworks acknowledge that environments shape behaviour or that motivation alone is insufficient to explain action. The dietary affordance framework builds on these contributions but differs in a specific respect: it provides a formally grounded selection mechanism that specifies how environmental properties are integrated with motivation and habit at the point of action selection, generating testable quantitative predictions. This supplementary note clarifies the relationship between the present framework and five prominent approaches.

**COM-B (Michie, Van Stralen, and West 2011).** The COM-B model identifies three necessary conditions for behaviour: Capability, Opportunity, and Motivation. Opportunity is explicitly structural, encompassing both physical opportunity (e.g., food availability, infrastructure) and social opportunity (e.g., norms, cultural practices). COM-B has been influential in intervention design and correctly positions environmental factors as determinants rather than mere moderators. However, COM-B functions as a diagnostic checklist: it identifies which conditions must be present but does not model how they interact to determine which action is selected when multiple options satisfy the minimum conditions. In a supermarket where both sustainable and unsustainable options are physically available, COM-B would classify opportunity as present for both. The dietary affordance framework goes further by modelling the relative affordance weight of competing options through the interaction of pragmatic value $V_{i,c}$, predictive precision $P_{i,c}$, and the policy prior $H_{i,c}$. This explains why one option can dominate even when all COM-B conditions are nominally met for both: the unsustainable option may have higher enactment precision and a stronger policy prior, giving it a higher relative affordance weight despite equivalent physical opportunity. COM-B identifies that opportunity matters; the present framework models how it competes.

**Self-Determination Theory (Deci and Ryan 2008).** SDT distinguishes between autonomous motivation (acting from genuine interest or internalised values) and controlled motivation (acting from external pressure or obligation) and predicts that behaviour is more likely to be sustained when motivation is autonomous. This distinction is important and well supported: a person who values sustainability for its own sake is, all else being equal, more likely to act on that value than one who feels externally pressured to do so. However, SDT's central prediction i.e., that autonomous motivation should be sufficient for sustained behaviour, encounters the same empirical problem as other intention-focused frameworks: many people report genuine, autonomously held sustainability values yet fail to enact them consistently. SDT can explain why motivation quality matters but does not specify why high-quality motivation still fails when the food environment is volatile, time-constrained, or dominated by high-precision alternatives. The dietary affordance framework addresses this directly. Even strongly autonomous motivation contributes to pragmatic value $V_{i,c}$, but if the sustainable policy carries low predictive precision $P_{i,c}$because prices are volatile, preparation is uncertain, or household acceptance is unpredictable, and lacks a competing policy prior $H_{i,c}$, it will be outweighed at the point of selection by familiar, high-precision alternatives whose policy priors have been built through years of repeated enactment. The framework therefore does not dispute SDT's claim that motivation quality matters but specifies the conditions under which even high-quality motivation is insufficient: when the affordance field does not support reliable enactment and no sustainable policy prior has been established.

**Ecological and socio-ecological models (Bronfenbrenner 1979; Sallis and Owen 2015).** Ecological models of health behaviour situate individual action within nested levels of influence: intrapersonal, interpersonal, organisational, community, and policy. These models have been important in establishing that behaviour cannot be explained by individual-level variables alone and that interventions should target multiple levels simultaneously. However, ecological models are architecturally descriptive rather than mechanistically specified. They identify where influence originates (which level of the system) but do not specify how influences from different levels are integrated at the point of action selection. A person may have strong individual motivation, supportive interpersonal norms, and community-level access to sustainable food, yet still select the processed option if the precision structure at the point of choice favours it. Ecological models cannot explain this outcome because they lack a formal account of how competing influences are weighted and resolved. The dietary affordance framework provides this account through the relative affordance weight, in which environmental reliability (a community- or policy-level variable) enters the same selection equation as personal preference (an intrapersonal variable) and habit strength (a learning-history variable). The framework is therefore compatible with ecological models but adds the selection mechanism they lack.

**Social practice theory (Shove, Pantzar, and Watson 2012).** Social practice theory offers the most radical departure from individual-behaviour frameworks. It dissolves "behaviour" into "practices", that is socially shared configurations of materials (objects, infrastructure), competences (skills, knowledge), and meanings (cultural significance, norms), that are reproduced through participation rather than chosen through individual decision-making. This perspective resonates with the present framework's emphasis on the constitutive role of environments: food practices persist not because individuals independently decide to repeat them, but because the material and social infrastructure continuously reproduces the conditions for their enactment. However, social practice theory is deliberately non-computational. By rejecting the individual selection frame, it forecloses the possibility of modelling when and why particular practices will shift, persist, or be displaced. It can describe the elements that sustain a practice but cannot generate quantitative predictions about the conditions under which a practice will change, for example, how much reduction in price volatility is needed before sustainable cooking becomes a stable practice, or whether a meat tax will produce substitution toward other unsustainable options rather than the intended shift. The dietary affordance framework preserves the relational and constitutive insights of social practice theory while embedding them in a formal selection mechanism capable of generating testable predictions (Table 1, main text).

**Choice architecture and nudge theory (Thaler, Sunstein, and Balz 2013).** The nudge tradition focuses on designing choice environments to steer behaviour toward preferred outcomes, typically by exploiting defaults, framing effects, and simplification. This shares the present framework's practical orientation toward environmental redesign. However, the theoretical basis of nudge theory is comparatively thin. Default effects are typically attributed to "status quo bias" or "inertia" i.e., descriptive labels that identify the phenomenon without specifying the underlying mechanism. The dietary affordance framework offers a mechanistic reinterpretation: defaults dominate not because of generic cognitive inertia, but because they function as high-precision policies with strong policy priors. A default option is easy to select (low interaction cost), has been selected before (strengthening $H_{i,c}$), and produces predictable outcomes (high $P_{i,c}$). This reinterpretation has a practical consequence that nudge theory does not anticipate. Nudge theory predicts that making the sustainable option the default should be sufficient to shift behaviour. The present framework predicts that defaults will hold only if the selected option is reliably executed after choice, that is, only if it delivers low post-choice surprise. A sustainable default that produces unexpected cost, poor satiety, or household rejection will rapidly lose predictive precision, causing the agent to actively override the default in subsequent encounters. The design standard therefore shifts from "make it the default" to "make it the default and ensure post-choice reliability," which is a substantively different intervention target.

The following table summarises these distinctions:

| **Framework** | **What it contributes** | **What the dietary affordance framework adds** |
| --- | --- | --- |
| COM-B | Identifies Opportunity as a necessary structural condition for behaviour | Models how opportunity competes with alternative options through relative affordance weight; explains why behaviour fails when opportunity is nominally present, but enactment precision is low relative to alternatives |
| Self-Determination Theory | Distinguishes motivation quality (autonomous vs. controlled) and predicts that autonomous motivation sustains behaviour | Specifies why even autonomous, genuinely held motivation fails when the affordance field does not support reliable enactment; locates the bottleneck in environmental precision and absent policy priors rather than motivation quality |
| Ecological models | Maps multiple levels of environmental influence on behaviour | Provides the selection mechanism by which influences from different levels are integrated and resolved at the point of action; explains how policy-level variables (price stability, infrastructure) enter the same decision equation as individual-level variables (preference, habit) |
| Social practice theory | Reconceptualises behaviour as socially reproduced practice rather than individual choice; emphasises the constitutive role of materials, competences, and meanings | Preserves the relational and constitutive insights while embedding them in a formal selection mechanism capable of generating testable, quantitative predictions about when practices will shift or persist |
| Choice architecture / nudge theory | Focuses on environmental design to steer behaviour through defaults and simplification | Provides a mechanistic account of why defaults work (high precision, strong policy prior) and predicts when they will fail (post-choice surprise degrades precision); shifts the design standard from "set the default" to "ensure post-choice reliability" |

**References**

Bronfenbrenner, U. (1979). *The ecology of human development: Experiments by nature and design*. Harvard University Press.

Deci, E. L., & Ryan, R. M. (2008). Self-determination theory: A macrotheory of human motivation, development, and health. *Canadian Psychology / Psychologie canadienne*, *49*(3), 182–185.

Michie, S., van Stralen, M. M., & West, R. (2011). The behaviour change wheel: A new method for characterising and designing behaviour change interventions. *Implementation Science*, *6*(1), 42.

Sallis, J. F., & Owen, N. (2015). Ecological models of health behavior. In K. Glanz, B. K. Rimer, & K. Viswanath (Eds.), *Health behavior: Theory, research, and practice* (5th ed., pp. 43–64). Jossey-Bass.

Shove, E., Pantzar, M., & Watson, M. (2012). *The dynamics of social practice: Everyday life and how it changes*. SAGE Publications.

Thaler, R. H., Sunstein, C. R., & Balz, J. P. (2013). Choice architecture. In E. Shafir (Ed.), *The behavioral foundations of public policy* (pp. 428–439). Princeton University Press.
