# Supplementary material for: From intention–behavior gap to dietary affordances: an active inference account of sustainable eating
Source: Front Psychol. 2026 Aug 11;17:1779626. doi: 10.3389/fpsyg.2026.1779626 (PMC13503352; doi:10.3389/fpsyg.2026.1779626)
Supplement: Supplementary file 2 [file Supplementary_file_2.docx]

**Supplementary Material 2**

**Mathematical Status and Decomposition of the Reduced-Form Policy Score in the Dietary Affordance Framework**

**Overview**

The main paper introduces **dietary affordance** as a relative selection weight shaped by pragmatic value $V_{i,c}$, predictive precision $P_{i,c}$, and the policy prior $H_{i,c}$. The reduced-form policy score is defined as:

$$S_{i,c}=V_{i,c}+P_{i,c}$$

where $i$denotes a dietary option or policy and $c$denotes the decision context.

This supplementary material has two purposes. The first is to clarify the mathematical status of this reduced form: which features of the equation are inherited directly from the variational free energy decomposition that underlies active inference, and which are deliberate empirical simplifications adopted to make the framework testable using observable environmental and behavioural variables.

The second is to expand predictive precision by decomposing $P_{i,c}$into architecturally distinct sources of uncertainty: financial, availability, temporal, skill-based, spoilage/storage, sensory/satiety, social, fallback, and interoceptive precision. This decomposition allows the framework to address how the same dietary option can be high-precision for one household and low-precision for another.

**Mathematical Status of the Reduced-Form Approximation**

In the standard active inference formulation (Friston, FitzGerald et al., 2016, 2017; Parr and Friston, 2019), the quantity that drives policy selection is **expected free energy**, denoted $G(\pi)$. For a given policy $\pi$, expected free energy decomposes into expected risk and expected ambiguity:

$$G(\pi)=\underset{\text{expected risk}}{\underset{⏟}{D_{\mathrm{KL}}\left[ Q(o\mid\pi)\text{ }\parallel\text{ }P(o) \right]}}+\underset{\text{expected ambiguity}}{\underset{⏟}{\mathbb{E}_{Q(s\mid\pi)}\left[ H\left( P(o\mid s) \right) \right]}}$$

where $Q(o\mid\pi)$is the predicted outcome distribution under policy $\pi$, $P(o)$ is the agent’s prior preference distribution over outcomes, $D_{\mathrm{KL}}$denotes Kullback–Leibler divergence, and $H\left( P\left( o \mid s \right) \right)$denotes the entropy of the likelihood mapping from hidden states $s$to observations $o$.

Both components are information-theoretic quantities measured in nats, and both enter $G(\pi)$ additively because they are derived from the same variational decomposition. In probability space, policies are selected through a softmax over the negative of expected free energy, weighted by a multiplicative policy prior $E(\pi)$, which captures accumulated tendencies to select particular courses of action independently of the current evaluative score.

The probability of selecting policy $\pi_{i}$can therefore be written as:

$$\Pr(\pi_{i})=\frac{E(\pi_{i})\exp\left[ -\gamma G(\pi_{i}) \right]}{\sum_{\pi_{j}\in\Pi} E(\pi_{j})\exp\left[ -\gamma G(\pi_{j}) \right]}$$

where $\gamma$is a precision or inverse-temperature parameter governing sensitivity to differences in expected free energy, and $\Pi$is the feasible policy set.

The reduced-form equation used in the main paper preserves this structural form but rewrites the score in positive-value terms. The unnormalised affordance weight of option $i$in context $c$is:

$$\tilde{A}_{i,c}=H_{i,c}\exp\left( \gamma_{c}S_{i,c} \right)$$

where:

$$S_{i,c}=V_{i,c}+P_{i,c}$$

The probability of selecting option $i$is obtained by normalising across the feasible affordance field $F_{c}$:

$$\Pr(\pi_{i}\mid c)=\frac{\tilde{A}_{i,c}}{\sum_{j\in F_{c}} \tilde{A}_{j,c}}$$

or, substituting the full expression:

$$\Pr(\pi_{i}\mid c)=\frac{H_{i,c}\exp\left[ \gamma_{c}S_{i,c} \right]}{\sum_{j\in F_{c}} H_{j,c}\exp\left[ \gamma_{c}S_{j,c} \right]}$$

The features inherited directly from the active inference formalism are:

1. the multiplicative role of the policy prior $H_{i,c}$, which approximates $E(\pi_{i})$in active inference notation;
2. the exponential transformation of the policy score;
3. the softmax normalisation across the feasible policy set;
4. the additive structure inside the exponent.

The additivity is not an arbitrary modelling choice. Inside the exponent of a softmax, the sum of two log-quantities corresponds to the product of two probability-like quantities. More importantly, the variational decomposition of expected free energy is itself additive in risk and ambiguity. Writing:

$$S_{i,c}=V_{i,c}+P_{i,c}$$

therefore inherits the mathematical structure of standard active inference policy selection. This is the formal basis of the framework.

What is not inherited, but assumed, concerns the specific empirical quantities being added. In the full formalism, expected risk is a Kullback–Leibler divergence between predicted and preferred outcome distributions, and expected ambiguity is the expected entropy of a likelihood mapping. Both are distributional quantities. They take into account not only mean differences between predicted and preferred states, but also the variance, skew, and uncertainty of those distributions.

The reduced-form equation collapses each into a scalar. $V_{i,c}$is treated as a scalar summary of expected preference fulfilment. $P_{i,c}$is treated as a scalar summary of expected enactment reliability. This collapse is necessary if the framework is to be parameterised from observable proxies such as price volatility, stock-out rates, preparation-time variance, household acceptance risk, or repeat-purchase frequency, because these proxies do not deliver full posterior distributions over outcomes. However, the collapse is a deliberate simplification and introduces specific limitations.

Two simplifications matter most for empirical interpretation.

**Simplification 1: Scalar Pragmatic Value** $\boldsymbol{V}_{\boldsymbol{i,c}}$

As defined in the main paper, $V_{i,c}$captures the expected alignment between an option’s outcomes and the agent’s prior preferences. In the full formalism, the corresponding expected risk term carries distributional information. Two options can produce identical mean alignment with prior preferences while differing in the variance, skew, or downside risk of their predicted outcomes. These would be evaluated differently in a full expected free energy model because the Kullback–Leibler divergence is sensitive to the full shape of the distribution, not only its mean.

The scalar reduction of $V_{i,c}$cannot represent this distinction.

Empirically, this matters when agents face asymmetric downside risk. For example, one sustainable meal may usually be satisfying but occasionally fail badly because the recipe is unfamiliar and does not deliver expected satiety. Another option may be consistently mediocre but never produce severe outcomes. In a fully specified model, these options would carry different expected risk. In the reduced form, they could be assigned the same $V_{i,c}$.

Future empirical work could test whether distributional features of outcome alignment, particularly variance and downside risk, predict choice above and beyond mean value. If they do, this would indicate that the scalar reduction is masking systematic structure rather than collapsing irrelevant detail.

**Simplification 2: Scalar Predictive Precision** $\boldsymbol{P}_{\boldsymbol{i,c}}$

Predictive precision in the main paper is defined as the inverse of combined uncertainty associated with executing a policy, and it is treated as a single scalar. In the full active inference formalism, however, what we are calling predictive precision is not a single quantity. It is a composite of several architecturally distinct sources of uncertainty that contribute to expected free energy through different routes.

Outcome ambiguity, the entropy of the likelihood mapping from hidden states to observations, contributes directly to expected free energy as the ambiguity term. Transition uncertainty, or the reliability with which actions produce intended state changes, is encoded in the $B$matrix of a discrete state-space generative model. It contributes through the entropy of state transitions and shapes both the predicted outcome distribution and the agent’s epistemic value. External volatility (price fluctuation, stock instability, spoilage risk, household acceptance variability) feeds into the agent’s beliefs about state transitions but is itself an environmental property rather than a precision parameter in the formal sense.

Treating these as elements of a single scalar $P_{i,c}$collapses architecturally distinct objects into a composite. This simplifies estimation but blurs the distinction between sources of uncertainty that respond to different interventions. A subsidy reduces price volatility but does not address transition uncertainty. A meal kit reduces transition uncertainty but may leave price volatility intact. Eco-labelling addresses outcome ambiguity by clarifying preference-relevant attributes but does not affect transition reliability. The scalar reduction obscures these distinctions.

A further consequence of this composite treatment is that the constituent sources of uncertainty are not all in the same units. Expected ambiguity is measured in nats. Price coefficient of variation is dimensionless. Preparation-time variance is measured in minutes squared. Stock-out frequency is a probability. Household acceptance risk may be binary or probabilistic. To combine these into a single scalar $P_{i,c}$, the reduced-form approach implicitly requires a standardisation step that converts heterogeneous proxies into commensurable quantities.

In practice, this may involve z-scoring within sample:

$$z_{i,c}^{\left( k \right)}=\frac{x_{i,c}^{\left( k \right)}-\mu^{\left( k \right)}}{\sigma^{\left( k \right)}}$$

where $x_{i,c}^{\left( k \right)}$ is the raw value of uncertainty or precision dimension $k$, and $\mu^{\left( k \right)}$and $\sigma^{\left( k \right)}$are parameters of the relevant reference distribution.

Alternatively, proxies may be mapped to a bounded common scale:

$$p_{i,c}^{\left( k \right)}\in[0,1]$$

where higher values represent greater precision. Dimension-specific weights may also be estimated through a procedure that calibrates the relative behavioural impact of different uncertainty sources against observed selection probabilities.

The choice of standardisation affects parameter estimates and should be reported transparently in any empirical application of the framework. This is not unique to the present model. Every reduced-form approximation of active inference that uses observable proxies must address commensurability. However, the requirement is worth stating explicitly.

The reduced-form equation is therefore best understood as a structurally faithful but distributionally simplified approximation of active inference policy selection. It preserves the multiplicative prior, exponential transformation, softmax normalisation, and additive policy score that the full formalism specifies. It collapses the distributional richness of expected risk and the architectural distinctness of uncertainty sources into scalars. It is justified as a working empirical scaffold for testing the framework’s qualitative predictions, not as a substitute for a fully specified discrete state-space generative model.

The final section of this supplement identifies the conditions under which the reduced form will diverge most strongly from a fuller treatment and where, accordingly, finer-grained decompositions should be introduced.

**Decomposing Predictive Precision**

With the formal status of the reduced form clarified, predictive precision can now be decomposed into dimension-specific sources of uncertainty, each of which can vary independently across dietary options and contexts.

For empirical purposes, predictive precision can be expressed as a context-weighted sum across $K$dimensions:

$$P_{i,c}=\sum_{k=1}^{K} w_{k,c}p_{i,c}^{\left( k \right)}$$

subject to:

$$\sum_{k=1}^{K} w_{k,c}=1$$

and:

$$w_{k,c}\geq0\forall k$$

where $p_{i,c}^{\left( k \right)}$ denotes the standardised precision of option $i$in context $c$along dimension $k$, and $w_{k,c}$denotes the context-specific weight assigned to that source of uncertainty.

The constraint that the weights sum to one ensures that the decomposed $P_{i,c}$remains on the same scale as the unitary $P_{i,c}$used in the main paper, so that the reduced-form policy score retains its original interpretation.

This formulation preserves the reduced-form role of $P_{i,c}$while making explicit that predictive precision is a context-weighted architecture of uncertainty sources. It also makes explicit that the additive combination of dimensions is itself a simplification, parallel to the additive combination of $V_{i,c}$and $P_{i,c}$at the policy-score level.

Some dimensions of precision may interact rather than combine additively. Low fallback precision, for example, may magnify the behavioural impact of low financial precision because the household lacks a buffer against failed or wasted purchases. Such interactions could be modelled by introducing dimension-specific interaction terms:

$$P_{i,c}=\sum_{k=1}^{K} w_{k,c}p_{i,c}^{\left( k \right)}+\sum_{k<l} w_{kl,c}p_{i,c}^{\left( k \right)}p_{i,c}^{\left( l \right)}$$

where $w_{kl,c}$captures the interaction between precision dimensions $k$and $l$in context $c$.

This interaction form increases estimation complexity and may create identifiability problems, especially in small samples. For most empirical applications, the additive context-weighted form provides an adequate first approximation. The divergence-conditions section identifies cases where interaction terms are likely to be necessary.

Under food insecurity, financial, spoilage/storage, and fallback precision may dominate because uncertainty carries high failure costs. In less constrained households, sensory/satiety, social, or other precision dimensions may play a larger role in determining whether a sustainable option is repeatedly selected. The context-specific weights $w_{k,c}$therefore carry substantive information about which sources of uncertainty are behaviourally consequential for whom, and their estimation is itself an empirical objective rather than a nuisance parameter.

If nine precision dimensions are specified, predictive precision can be written as:

$$\begin{matrix} P_{i,c}= & \text{ }w_{\mathrm{fin},c}p_{i,c}^{\left( \mathrm{fin} \right)}+w_{\mathrm{avail},c}p_{i,c}^{\left( \mathrm{avail} \right)}+w_{\mathrm{time},c}p_{i,c}^{\left( \mathrm{time} \right)}+w_{\mathrm{skill},c}p_{i,c}^{\left( \mathrm{skill} \right)} \\ & +w_{\mathrm{spoil},c}p_{i,c}^{\left( \mathrm{spoil} \right)}+w_{\mathrm{sens},c}p_{i,c}^{\left( \mathrm{sens} \right)}+w_{\mathrm{soc},c}p_{i,c}^{\left( \mathrm{soc} \right)}+w_{\mathrm{fall},c}p_{i,c}^{\left( \mathrm{fall} \right)}+w_{\mathrm{int},c}p_{i,c}^{\left( \mathrm{int} \right)} \end{matrix}$$

where the dimensions correspond to financial, availability, temporal, skill, spoilage/storage, sensory/satiety, social, fallback, and interoceptive precision.

This decomposition means that an option can have high pragmatic value but low predictive precision because one or more uncertainty dimensions make enactment unreliable. A plant-based meal may be financially predictable but socially uncertain if household acceptance is low, or affordable and available but uncertain in preparation time for a household operating under acute time pressure.

**Supplementary Table S1. Candidate Dimensions of Predictive Precision**

| **Dimension of predictive precision** | **What is uncertain?** | **Example low-precision sustainable option** | **Possible empirical proxy** |
| --- | --- | --- | --- |
| Financial precision | Whether the option will remain within budget | Fresh ingredients with variable prices | Price volatility, perceived affordability, budget overrun risk |
| Availability precision | Whether the option will be stocked or accessible when needed | Plant-based staples unavailable in local shops | Stock-out rates, distance to retailer, transport access |
| Temporal precision | Whether the option can be completed within available time | Cooking legumes or unfamiliar meals after work | Preparation-time variance, time scarcity, schedule instability |
| Skill / transition precision | Whether the individual can reliably execute the required action sequence | New recipes requiring unfamiliar techniques | Cooking confidence, recipe failure rate, equipment access |
| Spoilage / storage precision | Whether ingredients will remain usable until consumption | Fresh produce bought for planned meals | Spoilage rates, refrigeration access, household food waste |
| Sensory / satiety precision | Whether the meal will taste acceptable and provide expected fullness | Novel plant-based alternatives with uncertain taste or satiety | Liking ratings, expected satiety, satiety confidence, repeat purchase |
| Social precision | Whether others in the household or social setting will accept the option | A sustainable meal that children or partners may reject | Household rejection risk, meal conflict, need for separate meals |
| Fallback precision | Whether failure can be safely absorbed | Trying a new meal with no backup food or money | Availability of backup meals, financial buffer, food security status |
| Interoceptive precision | Whether the individual can reliably detect, predict, and contextualise internal hunger and satiety cues | Treated separately; developed in detail elsewhere | Treated separately; developed in detail elsewhere |

**Incorporating Decomposed Predictive Precision into the Policy Score**

Substituting the decomposed predictive precision term into the reduced-form policy score gives:

$$S_{i,c}=V_{i,c}+\sum_{k=1}^{K} w_{k,c}p_{i,c}^{\left( k \right)}$$

The relative affordance weight of option $i$in context $c$can therefore be written as:

$$\tilde{A}_{i,c}=H_{i,c}\exp\left[ \gamma_{c}\left( V_{i,c} + \sum_{k=1}^{K} w_{k,c}p_{i,c}^{\left( k \right)} \right) \right]$$

The probability of selecting option $i$is obtained by normalising across the feasible affordance field $F_{c}$:

$$\Pr(\pi_{i}\mid c)=\frac{\tilde{A}_{i,c}}{\sum_{j\in F_{c}} \tilde{A}_{j,c}}$$

or, substituting the full expression:

$$\Pr(\pi_{i}\mid c)=\frac{H_{i,c}\exp\left[ \gamma_{c}\left( V_{i,c} + \sum_{k=1}^{K} w_{k,c}p_{i,c}^{\left( k \right)} \right) \right]}{\sum_{j\in F_{c}} H_{j,c}\exp\left[ \gamma_{c}\left( V_{j,c} + \sum_{k=1}^{K} w_{k,c}p_{j,c}^{\left( k \right)} \right) \right]}$$

This expression makes explicit that the probability of selecting a dietary policy depends on its pragmatic value, the context-weighted reliability with which it can be enacted across distinct sources of uncertainty, and the strength of the learned policy prior. Each component carries its own simplification, and each can in principle be unpacked further as data and modelling capacity permit.

**When the Reduced Form Will Diverge from a Fuller Treatment**

The reduced-form approximation is justified for the empirical purposes of the main paper but will diverge from a fully specified active inference generative model under specific conditions. Identifying these conditions in advance allows researchers to anticipate where the framework will be most informative and where finer-grained decompositions should be introduced.

Four conditions are particularly relevant.

First, divergence is expected where outcome distributions are substantially asymmetric. The scalar $V_{i,c}$captures mean alignment between predicted and preferred outcomes but is insensitive to variance, skew, and downside risk. When the choice involves options whose predicted outcome distributions differ in variance or shape (for example, a usually satisfying but occasionally failing sustainable meal versus a consistently mediocre processed alternative) the reduced form will misestimate the relative attractiveness of the options.

In such cases, a fuller treatment using the Kullback–Leibler divergence between predicted and preferred outcome distributions, with explicit representation of variance and downside risk, is likely to provide better empirical fit. This is most likely to matter under high constraint, where the cost of severe negative outcomes is asymmetric to the benefit of average positive outcomes.

Second, divergence is expected where uncertainty sources within $P_{i,c}$respond differently to the same intervention. The composite scalar $P_{i,c}$collapses outcome ambiguity, transition uncertainty, and external volatility into a single quantity. When an intervention targets one source while leaving others intact (for example, a subsidy that reduces financial uncertainty without addressing skill or social uncertainty, or a meal kit that reduces transition uncertainty without addressing price volatility) the scalar $P_{i,c}$will mis-predict the magnitude of the behavioural response.

The decomposed $P_{i,c}$ with dimension-specific weights addresses this directly by allowing intervention effects to be estimated against the dimension they target. Where interventions target multiple dimensions simultaneously, or where dimensions interact such that low precision in one amplifies the impact of low precision in another (for example, low fallback precision amplifying the behavioural impact of low financial precision because the household lacks a buffer against failed or wasted purchases) additional interaction terms may be required.

Third, divergence is expected where context inference itself is non-trivial. Both $H_{i,c}$and $\gamma_{c}$ are written as context-dependent quantities, but the framework treats context as given. In practice, agents must infer which context they are in before retrieving context-appropriate priors and precision weights. A person may behave differently in their kitchen at home, in a workplace canteen, in a friend’s house, and in a supermarket near closing time, even when objective options are similar, because they are inferring different contexts and retrieving different policy priors.

Hierarchical active inference (Pezzulo, Rigoli et al., 2018) provides apparatus for modelling this, but it requires specifying how contexts are represented, how context inference proceeds, and how policy priors are structured across contexts. The present framework leaves this architecture implicit, which is appropriate for a reduced form but limits its applicability where context-shifting is the central phenomenon.

Fourth, divergence is expected where belief-updating dynamics are central to the question. The static formulation treats $V_{i,c}$, $P_{i,c}$, and $H_{i,c}$as quantities at a moment in time. The temporal extension introduced in the main paper for the initiation problem makes these time-varying but does not specify the learning dynamics that govern their evolution.

A fully specified active inference model would represent belief updating through Bayesian inference and Dirichlet concentration parameters that accumulate evidence with each successful or failed enactment. Where the empirical question concerns the rate of habit formation, the number of successful encounters needed to establish a competing policy prior, or the conditions under which a single negative experience durably suppresses future selection, the reduced form should be extended with explicit belief-updating dynamics.

These four conditions identify the boundaries within which the reduced-form approximation is most defensible: relatively symmetric outcome distributions, interventions whose effects are not strongly differential across uncertainty dimensions, stable contexts that the agent has already learned to identify, and questions about steady-state selection rather than learning dynamics.

Within these boundaries, the reduced form provides an empirically tractable scaffold that preserves the qualitative structure of active inference policy selection while remaining estimable from observable variables. Outside these boundaries, the framework should be extended with the relevant fuller treatment, with the reduced form serving as a starting point rather than a final specification.

**Implications for Empirical Testing**

The decomposition and the explicit identification of formal status together generate several empirical advantages.

First, they prevent predictive precision from functioning as a broad residual category for any form of difficulty or constraint. Each dimension is specified, measurable, and intervention-relevant.

Second, they allow researchers to test which sources of uncertainty most strongly influence dietary policy selection in different contexts, and to estimate context-specific weights $w_{k,c}$rather than treating them as uniform.

Third, they make inequality central to the model: the same uncertainty source may be weighted differently depending on household resources, fallback capacity, food security, time availability, and social context.

Fourth, they specify the conditions under which the reduced form will be insufficient and a fuller treatment will be required, making the framework’s limitations diagnosable rather than diffuse.

A small risk of ingredient spoilage may have minimal behavioural effect for a household with financial slack and backup food. The same risk may strongly reduce selection probability for a household experiencing food insecurity because failure cannot be safely absorbed. In the model, this difference is represented not only by lower spoilage/storage precision but by a higher context-specific weight assigned to that dimension of uncertainty:

$$w_{\mathrm{spoil},c}^{food insecure}>w_{\mathrm{spoil},c}^{food secure}$$

The decomposition therefore strengthens the framework’s claim that dietary behaviour is not simply a function of individual motivation or objective food availability. Behaviour emerges from the interaction between values, uncertainty architecture, household constraint, and learned policy priors, with the additive reduced form providing a structurally faithful first approximation that can be extended where the conditions of its validity are not met.

**References**

Friston, K., FitzGerald, T., Rigoli, F., Schwartenbeck, P., O'Doherty, J., and Pezzulo, G. (2016). Active inference and learning. *Neuroscience and Biobehavioral Reviews, 68*, 862–879.

Friston, K., FitzGerald, T., Rigoli, F., Schwartenbeck, P., and Pezzulo, G. (2017). Active inference: a process theory. *Neural Computation, 29*(1), 1–49.

Parr, T., and Friston, K. J. (2019). Generalised free energy and active inference. *Biological Cybernetics, 113*(5–6), 495–513.

Pezzulo, G., Rigoli, F., and Friston, K. J. (2018). Hierarchical active inference: a theory of motivated control. *Trends in Cognitive Sciences, 22*(4), 294–306.
